# Host–Microbiota Interactions in the Regulation of Intestinal Health in Weaned Piglets: Molecular Mechanisms and Nutritional Intervention Strategies

**DOI:** 10.3390/ani16142117

**Published:** 2026-07-08

**Authors:** Tiantian Li, Runan Zhang, Qianqian Gao, Jiajing Chang, Xiaonan Zeng, Xianlong Feng, Zhanxiang Zhang, Siyu Wei, Chunlan Xu, Lei Qiao

**Affiliations:** 1 School of Life Science and Technology, Northwestern Polytechnical University, Xi’an 710000, China; tianli@mail.nwpu.edu.cn (T.L.); zhangrunan@mail.nwpu.edu.cn (R.Z.); gq16@mail.nwpu.edu.cn (Q.G.); changjj@mail.nwpu.edu.cn (J.C.); zxnobey@mail.nwpu.edu.cn (X.Z.); xianlongfeng@mail.nwpu.edu.cn (X.F.); zzx1@mail.nwpu.edu.cn (Z.Z.); clxu@nwpu.edu.cn (C.X.); 2 Key Laboratory of Molecular Animal Nutrition, Ministry of Education, College of Animal Sciences, Zhejiang University, Hangzhou 310058, China; sywei@zju.edu.cn

**Keywords:** weaned piglets, gut microbiota, host–microbiota interaction, intestinal barrier, precision nutrition, antibiotic-replacement strategies

## Abstract

The weaning period represents a critical developmental window during which the intestinal host–microbiota interaction network in piglets undergoes profound remodeling and becomes highly vulnerable to disruption. Abrupt dietary transition, reduced feed intake, environmental stressors, and pathogen exposure can collectively interfere with gut microbial succession, epithelial barrier maturation, mucosal immune homeostasis, and neuroendocrine regulation, thereby increasing the susceptibility of piglets to post-weaning diarrhea, intestinal inflammation, and growth retardation. Gut microorganisms contribute to intestinal homeostasis by regulating epithelial renewal, barrier function, immune tolerance, inflammatory thresholds, and host metabolic adaptation through diverse microbial-derived signals, including short-chain fatty acids, tryptophan metabolites, bile acid derivatives, and amino acid-derived metabolites. Conversely, the host actively shapes microbial composition, spatial organization, and functional activity through mucins, antimicrobial peptides, secretory immunoglobulin A, intestinal alkaline phosphatase, epithelial oxygen gradients, and genetic factors. Dysregulation of these bidirectional interactions under weaning stress may shift the intestinal ecosystem from a homeostatic state toward dysbiosis and inflammation. In this review, we summarize current knowledge regarding the establishment and succession of the piglet gut microbiota, the molecular mechanisms underlying host–microbiota crosstalk, the disruption of these interaction networks during weaning stress, and the application of multi-omics approaches to decipher causal mechanisms. We further discuss microbiota-targeted nutritional strategies, including probiotics, prebiotics, synbiotics, postbiotics, functional amino acids, trace elements, and bioactive compounds, for improving intestinal health in weaned piglets. This review aims to provide a mechanistic framework for precision nutritional regulation of piglet intestinal health and for the development of antibiotic-alternative strategies in swine production.

## Simple Summary

Piglets are especially vulnerable to intestinal disturbance after weaning because diet, environment, stress, and pathogen exposure change at the same time. This review summarizes how gut microorganisms and host intestinal tissues communicate through microbial metabolites, barrier molecules, immune signals, and neuroendocrine pathways. It also outlines how microbiota-targeted nutritional strategies may help restore intestinal homeostasis and support antibiotic-replacement approaches in pig production.

## 1. Research Background

In modern intensive pig production systems, piglet intestinal health is closely associated with post-weaning survival, growth performance, feed efficiency, and disease prevention and thus represents a key determinant of production stability during the nursery stage. Post-weaning diarrhea, reduced feed intake, growth retardation, and secondary infections decrease production efficiency and increase the use of antimicrobials and treatment costs. In previous production systems, antibiotics and high-dose zinc oxide were commonly used to reduce weaning-related risks. However, with the progressive implementation of antibiotic-reduction policies and the increasing demand for antibiotic-alternative strategies, intestinal health management in piglets should move beyond conventional antibacterial and antidiarrheal approaches. Instead, greater emphasis should be placed on the coordinated regulation of the gut microbiota, barrier function, mucosal immunity, and nutritional metabolism during the critical weaning window [1,2,3,4].

### 1.1. Production Relevance of Piglet Intestinal Health

The weaning period is one of the most vulnerable stages in pig production, characterized by pronounced fluctuations in intestinal health and growth performance. After weaning, piglets commonly exhibit reduced feed intake, increased diarrhea incidence, impaired growth performance, and decreased within-batch body-weight uniformity. These problems compromise nursery survival and feed efficiency, thereby increasing treatment costs and management pressure [2].

After separation from the sow, piglets experience an abrupt interruption of milk supply, and their diet shifts from milk-derived nutrients to solid feed mainly composed of cereals and plant proteins. At the same time, changes in the rearing environment, mixing with unfamiliar piglets, and increased pathogen exposure often occur simultaneously. During this period, the intestinal barrier structure, digestive enzyme secretion, mucus layer defense, and mucosal immune system of piglets are not yet fully mature [1,3]. As a result, their capacity to adapt to nutritional and environmental changes remains limited, making the weaning period a high-risk stage for intestinal injury and diarrhea.

In the context of antibiotic reduction, intestinal health management in weaned piglets faces increasing pressure. For many years, post-weaning diarrhea has been controlled mainly with antimicrobial agents and pharmacological doses of zinc oxide. However, stricter restrictions on antibiotic and zinc oxide use have limited the sustainability of this conventional approach. Several emerging strategies, including phage therapy, engineered probiotics, postbiotics with defined dose-response relationships, fecal microbiota transplantation (FMT), and CRISPR-mediated microbiota editing, are now being explored as potential alternatives. Although most of these approaches remain experimental or at an early translational stage in pig production, they provide useful directions for developing more precise and mechanism-based strategies to maintain intestinal homeostasis in weaned piglets.

However, with the more standardized use of antimicrobials and increasingly strict restrictions on zinc oxide application, this conventional approach can no longer fully meet the current requirements for safety and sustainability in pig production. More importantly, intervention strategies centered solely on pathogen suppression have inherent limitations. On the one hand, a reduction in pathogen load does not necessarily indicate recovery of the disrupted intestinal ecosystem after weaning. Even when pathogen numbers are temporarily controlled, dysbiosis of the gut microbiota, reduced production of beneficial microbial metabolites, insufficient epithelial barrier repair, and sustained mucosal immune activation may still persist. These unresolved disturbances may partly explain the recurrence of diarrhea and the unstable recovery of growth performance after the withdrawal of antimicrobial or antidiarrheal treatments. On the other hand, broad-spectrum antimicrobial pressure may further disturb commensal microbial communities, weaken colonization resistance and beneficial fermentation functions, and impose selective pressure on antimicrobial resistance-associated genes or resistant strains.

Therefore, current regulation of piglet intestinal health should shift from the simple suppression of pathogenic bacteria toward maintaining gut microbial stability, promoting intestinal barrier repair, and improving host adaptive capacity [4].

### 1.2. Fundamental Role of the Early Gut Microbiota

During early development, the gut microbiota provides an important biological foundation that links nutrient utilization, barrier development, and immune maturation in piglets. The gut microbiota can degrade milk- or plant-derived nutritional substrates and participates in the production of short-chain fatty acids (SCFAs), bile acid transformation, and vitamin synthesis. It can also restrict the colonization of potential pathogens through niche competition. In addition, microbiota-derived metabolites and microbial-associated molecules can act on intestinal epithelial cells, the mucosal immune system, and neuroendocrine pathways, thereby influencing the functional development of the piglet intestine [5]. After birth, the gut microbiota gradually shifts from an early-colonized, low-complexity community toward a mature microecological system. This process spans key stages including birth, suckling, creep feeding, and the transition at weaning. Early microbial colonization is strongly shaped by maternal and perinatal factors. Delivery mode determines the first microbial exposures of newborn piglets, including contact with the sow reproductive tract, feces, skin, and the farrowing environment. After birth, colostrum and milk further provide both microbial and non-microbial signals that influence intestinal colonization. In particular, milk-derived secretory immunoglobulin A (sIgA) can bind selected microorganisms and microbial antigens, thereby helping regulate bacterial adhesion, immune exclusion, and the establishment of mucosal tolerance. Milk oligosaccharides, especially porcine milk oligosaccharides (PMOs), may also act as selective substrates for beneficial bacteria and as decoy receptors that limit pathogen attachment. Therefore, stable early colonization should not be viewed only as passive microbial acquisition, but as a coordinated process shaped by delivery-associated seeding, colostrum intake, milk immune factors, milk glycans, and the immature intestinal niche [6,7,8].

Whether early microbial colonization is stable and orderly affects not only the development of intestinal epithelial barrier structure and function but also the establishment of mucosal immune tolerance, the formation of nutrient-metabolic capacity, and the subsequent resistance of piglets to pathogen infection and weaning stress. This process is particularly sensitive to the quality and timing of colostrum intake because colostrum supplies maternal immunoglobulins, growth factors, bioactive proteins, microbial components, and milk glycans that jointly influence epithelial maturation and early microbiota assembly.

The intestine is not only responsible for nutrient digestion and absorption but also serves as an important ecological interface through which the gut microbiota continuously exchanges signals with the host immune, nervous, and endocrine systems. The gut microbiota interacts with epithelial barrier function, mucosal immunity, and metabolic regulatory pathways through microbe-derived signaling molecules such as SCFAs, tryptophan metabolites, bile acid derivatives, and amino acid-derived metabolites [9,10]. Conversely, the host regulates microbial composition, spatial distribution, and functional status by secreting mucins, antimicrobial peptides, sIgA, bile acids, and intestinal alkaline phosphatase [11]. Within this interaction network, the mucosal immune system performs signal recognition and response regulation, discriminating commensal-associated signals from potential danger signals and determining whether intestinal responses tend toward immune tolerance or inflammatory activation. At the same time, the gut–brain axis transmits local intestinal microecological disturbances to feeding regulation, stress responses, and systemic metabolic processes through neuroendocrine and immune-mediated pathways. Studies indicate that the microbial interaction network is particularly vulnerable during weaning. After weaning stress occurs, piglets often first show a transient decrease in feed intake, which may subsequently be accompanied by loose feces, reduced vitality, slower growth, and decreased feed conversion efficiency [6,12].

### 1.3. Pigs as a Model for Host–Microbiota Interactions

Pigs are important production animals in modern animal husbandry and also represent a valuable large-animal model for studies of host–microbiota interactions. Compared with rodents, pigs share greater similarities with humans in gastrointestinal anatomy, omnivorous feeding behavior, intestinal development, immune characteristics, and nutrient metabolism [13]. In addition, compared with single-factor experimental models, the piglet weaning model more closely reflects the dynamic disruption of host–microbiota interaction networks under complex physiological stress and therefore has greater production relevance and physiological complexity [5].

Metagenomic studies further support the value of pigs as a model for host–microbiota interaction research from the perspective of microbial functional capacity [14]. Xiao (2016) established a pig gut microbial gene catalog and found that it covered approximately 96% of the functional pathways present in the human gut microbiome [13]. This indicates a high degree of similarity between the porcine and human gut microbiomes at the level of functional potential, although this similarity does not imply identical species composition or strain-level correspondence [15].

Based on these physiological and substantial similarities to humans in gastrointestinal structure and function, the pig model has both practical production significance and translational biomedical value. The weaning period in piglets involves concurrent dietary transition, environmental stress, microbial community restructuring, and host physiological adaptation, making it a critical window for investigating host–microbiota interactions and the mechanisms underlying intestinal homeostasis [16,17].

This review focuses on weaned piglets and summarizes current knowledge regarding the establishment and succession of the intestinal microbiota, the bidirectional regulation mediated by microbial metabolites and host signaling networks, the disruption of host–microbiota interaction networks under weaning stress, and recent advances in the application of multi-omics approaches and nutritional interventions for mechanistic studies [18,19]. To facilitate accurate interpretation of the available evidence, studies derived from other animal models, human populations, or in vitro systems are clearly distinguished throughout the review, and their scope of applicability is discussed where relevant, thereby avoiding the direct extrapolation of non-piglet findings to weaned piglets.

## 2. Materials and Methods

This narrative review summarizes current evidence on host–microbiota interactions involved in the regulation of intestinal health in weaned piglets. The review focuses on microbial colonization and succession, epithelial barrier development, mucosal immune regulation, microbial metabolites, neuroendocrine communication, weaning-associated dysbiosis, omics-based approaches, and microbiota-targeted nutritional strategies.

A structured literature search was performed using PubMed, Web of Science, Scopus, and Google Scholar [20]. The final search was completed on 31 May 2026. The search mainly covered publications from 2010 to 2026, as this period includes most studies applying high-throughput sequencing, metagenomics, metabolomics, and other omics-based methods in piglet gut microbiota research. Earlier studies were also considered when they provided foundational information on intestinal barrier development, mucosal immunity, microbial colonization, or host–microbiota communication.

The search strategy combined terms related to the target animal, intestinal microbiota, weaning stress, host responses, microbial metabolites, omics technologies, and nutritional interventions. Boolean operators were used to construct the search strings. The main terms included: “piglets”, “weaned piglets”, “weaning piglets”, “weaning stress”, “post-weaning diarrhea”, “gut microbiota”, “intestinal microbiota”, “microbiome”, “host-microbiota interaction”, “host-microbe interaction”, “microbial colonization”, “microbiota succession”, “early-life microbiota”, “intestinal barrier”, “tight junction”, “mucosal immunity”, “sIgA”, “SCFAs”, “tryptophan metabolites”, “bile acids”, “amino acid metabolism”, “gut-brain axis”, “neuroendocrine”, “HPA axis”, “metagenomics”, “metabolomics”, “single-cell transcriptomics”, “multi-omics”, “probiotics”, “prebiotics”, “synbiotics”, “postbiotics”, “fecal microbiota transplantation”, “functional amino acids”, “crude protein reduction”, “low-protein diet” and “reduced-protein diet”. The detailed search strings and the number of records retrieved from each database are shown in Table 1.

All retrieved records were exported and checked for duplication. After removing duplicates, the remaining records were screened by title and abstract. Articles clearly unrelated to piglet intestinal health, gut microbiota, host–microbiota interactions, or nutritional regulation were excluded. Potentially eligible studies were then assessed by full-text review. In addition, the reference lists of key articles and recent reviews were manually examined to identify relevant studies not captured by the initial database search.

Studies were included if they investigated gut microbial colonization, succession, or dysbiosis in piglets; examined associations between the gut microbiota and epithelial barrier function, mucosal immunity, microbial metabolites, or neuroendocrine signaling; evaluated the effects of weaning stress on intestinal health, diarrhea, inflammation, metabolism, or growth performance; applied omics or multi-omics approaches to host–microbiota interactions; or assessed microbiota-targeted interventions relevant to piglet intestinal health.

Studies were excluded if they were not directly related to intestinal health, host–microbiota interactions, or piglet production. Articles focusing only on non-intestinal traits without mechanistic relevance to gut function, lacking sufficient methodological information, or without accessible full text were also excluded. Non-peer-reviewed sources were generally excluded, except for authoritative guidelines, consensus papers, or book chapters used to clarify definitions or background concepts. Studies based on rodents, humans, cell models, or in vitro systems were included only when they provided mechanistic evidence relevant to piglet intestinal physiology or host–microbiota interactions, and such evidence was interpreted cautiously rather than treated as a direct substitute for piglet-based data.

The study selection process followed a Preferred Reporting Items for Systematic Reviews and Meta-Analyses (PRISMA)-style workflow, including record identification, duplicate removal, title and abstract screening, full-text assessment, and final inclusion. Because this article was designed as a narrative review rather than a formal systematic review or meta-analysis, no protocol registration was performed and no quantitative evidence synthesis was conducted. Nevertheless, the search and screening procedures were structured to improve transparency and reproducibility. To further strengthen the critical evaluation of the available evidence, the included studies were interpreted according to the source of experimental evidence. Findings obtained directly from piglets or porcine intestinal models were considered the primary evidence base for this review. Evidence derived from rodent, human, cell, or in vitro studies was used only to support mechanistic interpretation when piglet-specific evidence was limited. In these cases, the evidence was described as indirect or extrapolated, and conclusions were presented with appropriate caution.

## 3. Establishment and Succession of the Piglet Gut Microbiota

The establishment and succession of the intestinal microbiota in piglets provide the ecological foundation for the development of host–microbiota interaction networks. Following birth, microorganisms originating from maternal, environmental, and dietary sources continuously colonize the gastrointestinal tract, and microbial communities gradually undergo selection, assembly, and stabilization within intestinal ecological niches. A stable early-life microbiota facilitates the adaptation of piglets to the transition from milk-based nutrition to solid-feed consumption. In contrast, factors such as antibiotic exposure, inadequate colostrum intake, environmental stress, and pathogen challenge may disrupt normal microbial colonization trajectories, thereby increasing the risk of intestinal dysfunction and impaired gut health during the weaning period [21,22,23,24,25].

### 3.1. From Colonization at Birth to Succession at Weaning

The establishment of the piglet gut microbiota begins around birth. The delivery process and the first hours to days after birth are the most active periods for microbial input, niche selection, and initial colonization. During parturition, piglets acquire initial microbial sources through contact with sow vaginal secretions, feces, skin, and the farrowing environment. After birth, colostrum, milk, teat skin, sow feces, and pen floors continue to provide multiple microbial inputs and jointly influence the composition and developmental trajectory of the early gut microbiota. Source-tracking studies have shown that maternal vaginal microorganisms make a substantial contribution to the early gut microbiota of newborn piglets, whereas the contributions from sow feces and the environment gradually increase thereafter. This process indicates that gut microbial establishment in piglets follows a degree of temporal order: maternal microorganisms provide early seeding, environmental microorganisms continue to supplement the community, and the host intestinal environment determines which bacteria can persist [26].

In early life, the piglet intestinal lumen remains relatively oxygen rich, allowing facultative anaerobes to occupy early niches and colonize first. As these early colonizers consume oxygen and alter the intestinal redox environment, the lumen gradually shifts toward hypoxic or anaerobic conditions, providing an ecological basis for the subsequent expansion of obligate anaerobes and increasing microbial complexity. This process drives the community from a low-complexity state toward a relatively stable anaerobic ecosystem. Longitudinal studies have divided fecal microbiota development from birth to 7 days post-weaning into three stages: birth to day 7, day 7 to weaning, and weaning to 7 days post-weaning. These findings suggest that piglet gut microbial development exhibits distinct temporal characteristics and stage-specific succession patterns [27].

Dietary substrate transition is a major driver of functional maturation and ecological remodeling of the piglet gut microbiota. During suckling, piglets mainly consume milk, and the gut microbiota is more oriented toward utilization of lactose, galactose, and milk-derived oligosaccharides. Metagenomic studies have shown that *Bacteroides* is relatively abundant in the fecal microbiota of suckling piglets, together with enrichment of genes related to lactose and galactose utilization. After weaning, the diet shifts to solid feed based mainly on cereals and plant proteins, and luminal substrates change from milk-derived sugars to plant polysaccharides, non-starch polysaccharides, and protein components. Correspondingly, taxa such as *Prevotella* and *Lactobacillus* increase in the fecal microbiota, and functions related to carbohydrate and amino acid metabolism are enhanced [25].

### 3.2. Major Factors Affecting Microbial Colonization

The establishment of the piglet gut microbiota is influenced by multiple maternal, environmental, host, and management-related factors. Maternal microbial transmission, colostrum quality, the farrowing environment, host genetic background, feeding management, early supplemental feeding, antibiotic exposure, and weaning age can all alter early colonization trajectories [26,28].

The sow contributes substantially to early piglet gut microbiota development through delivery-associated microbial transfer and through immune and nutritional factors provided by colostrum and milk. The immunoglobulins, milk-derived oligosaccharides, antimicrobial factors, and other bioactive substances they contain can influence early microbial colonization patterns and the formation of dominant microbial groups by regulating intestinal immune status, providing selective nutritional substrates, and limiting the expansion of potential pathogens. Maternal influence also changes with postnatal age. In early life, maternal microbial contributions are more pronounced. As piglets become more active, sow feces, the pen environment, and feed sources gradually contribute to shaping the microbiota [28].

Host genetic background also contributes to microbial community assembly. Associations have been identified between the porcine genome and gut microbial composition. Host genetic background determines the basic features of the intestinal microecological selective environment, whereas diet and feeding management further influence the direction of microbial structural and functional regulation within this environment. Microbiome-wide genome association studies (mGWAS) link host genotype with microbial abundance. Such studies have suggested that *Bacteroidales*_RF16_group may be regulated by host genetics, with candidate genes including EGF, ENPEP, and CASP6 [26].

Feeding management also affects the rate of microbial maturation. Early supplemental feeding before weaning, particularly exposure to diets containing dietary fiber or plant-derived nutritional substrates, can promote adaptive adjustment of the gut microbiota to the post-weaning feed-substrate environment. Early feeding may accelerate microbial maturation, mainly as reflected by increased microbial diversity and the pre-weaning appearance of some taxa typically associated with the post-weaning period [21,29].

Antibiotic exposure may interrupt the rhythm of early colonization. In addition to causing short-term microbial perturbations, early-life antibiotic treatment may produce persistent effects on intestinal gene expression, immune-related pathways, and microbial reconstruction after weaning. In a longitudinal pig study, antibiotic and stress treatments applied at day 4 after birth altered intestinal gene-expression profiles at day 55 and were associated with differences in gut microbiota composition at day 176, suggesting that early-life perturbations can influence the later developmental trajectory of the intestinal ecosystem [18]. Another longitudinal study following pigs from the pre-weaning period to the finishing stage showed that antimicrobial-use regimens shaped gut microbiota composition, immune parameters, and antimicrobial resistance gene profiles across developmental stages [19]. These findings indicate that antibiotic exposure during early life should not be regarded only as a transient disturbance. Rather, its effects may interact with weaning-associated microbial acquisition, immune programming, and environmental conditions to influence subsequent gut homeostasis. Therefore, although antibiotic intervention can reduce the load of some pathogenic microorganisms in the short term, excessive or poorly timed early use may disrupt normal maturation of the piglet gut microbiota, weaken functional redundancy and ecological resilience, increase selective pressure on the resistome, and alter adaptive responses to weaning stress [7,16].

### 3.3. Functional Characteristics of a Healthy Microbiota

The health status of the intestinal microbiota in piglets cannot be determined solely by an increase in a particular beneficial microorganism or a decrease in a specific potential pathogen. Changes in the abundance of individual taxa reflect only limited aspects of the microbial ecosystem and are insufficient to indicate the overall stability of the gut microbiota. A more comprehensive assessment should integrate multiple dimensions, including community structure, the composition of core microbiota, functional redundancy, microbial metabolite production, and host phenotypes related to intestinal barrier integrity and immune function. Among these features, functional redundancy is particularly important for ecosystem stability. It means that multiple microbial taxa can perform overlapping ecological or metabolic functions, such as fiber fermentation, short-chain fatty acid production, bile acid transformation, amino acid metabolism, or pathogen exclusion. When one taxon decreases after weaning stress, other taxa with similar functions may partially compensate for the lost activity, thereby helping maintain metabolic output and ecological resilience. Weaning stress may weaken this functional redundancy. Abrupt dietary transition, reduced feed intake, environmental stress, and pathogen exposure can reduce protective bacteria and disrupt microbial functions related to fermentation, nutrient metabolism, and mucosal protection. Under these conditions, the microbiota may lose its capacity to buffer external disturbances, making the ecosystem more susceptible to dysbiosis, pathogen expansion, reduced beneficial metabolite production, and inflammatory activation. Therefore, the health status of the piglet gut microbiota should not be judged only by taxonomic diversity or the abundance of individual genera. It should also be evaluated according to whether key functions are maintained by multiple microbial members and whether the microbiota can resist and recover from weaning-related perturbations [5,29].

Modern microbial ecology also emphasizes the distinction between the core microbiota and transient or passenger microbiota. The core microbiota refers to microbial members or functions that are consistently present across individuals, intestinal sites, or developmental time points and that contribute to stable host–microbiota interactions. In piglets, such core members may support substrate fermentation, mucus-layer adaptation, epithelial barrier maturation, immune tolerance, and colonization resistance. In contrast, passenger microbiota are more transient and are often introduced through feed, the environment, or short-term physiological disturbances. These taxa may fluctuate rapidly and may not necessarily contribute to long-term intestinal stability. Distinguishing core microbial members and core functions from passenger taxa is therefore essential for identifying microbial signatures associated with healthy development and for selecting reliable probiotic or microbiota-targeted intervention candidates.

Because functional redundancy and core microbiota cannot be fully resolved by taxonomic composition alone, multi-omics technologies are increasingly needed to evaluate the functional characteristics of the piglet gut microbiota. 16S rRNA sequencing can reveal microbial structure and changes in dominant taxa, but its explanatory power is largely limited to the taxonomic level. Metagenomic sequencing can further analyze functional genes and metabolic potential carried by microorganisms. Metabolomics can determine whether these functional potentials are translated into actual metabolite production. Integration with host transcriptomics, proteomics, or single-cell transcriptomics can further elucidate the response mechanisms of intestinal epithelial cells, immune cells, and related signaling pathways to microbial changes. This perspective is important for understanding the relationships among microbial dysbiosis, barrier injury, and immune abnormalities in weaned piglets. Post-weaning diarrhea usually involves multiple changes, including microbial functional remodeling, loss of ecological resilience, reduced barrier integrity, mucosal immune activation, and impaired metabolic adaptation. Integrative analysis of microbial composition, functional genes, metabolite profiles, and host response features is therefore necessary to determine whether weaning stress disrupts only community structure or also weakens core microbial functions and functional redundancy. This approach can help distinguish transient passenger taxa from stable core members, identify microbial functions that are consistently associated with intestinal health, and clarify how microbial dysbiosis is converted into barrier injury and immune imbalance in weaned piglets [3,6,15].

### 3.4. Long-Term Effects of Early Colonization

The effects of early gut microbial colonization show a degree of persistence and may influence subsequent immune development, metabolic maturation, and maintenance of intestinal homeostasis. The period from birth to weaning is a sensitive stage characterized by rapid development of the intestinal epithelium, mucosal immunity, and microbiota. Stable and orderly early colonization helps establish immune tolerance and promote mature barrier function, whereas disturbances such as antibiotic exposure, insufficient colostrum intake, environmental stress, or pathogen pressure may disrupt early colonization trajectories, weaken the foundation of intestinal homeostasis, and increase the burden of inflammatory responses and microbial recovery during weaning.

Epigenetic regulation provides a plausible explanation for how early microbial perturbations can produce persistent physiological effects. Intestinal microorganisms and their metabolites can affect DNA methylation, histone modification, and related processes, thereby altering the expression patterns of host genes and participating in long-term regulation of immune development, barrier function, and metabolic homeostasis. Short-chain fatty acids such as butyrate and propionate can inhibit histone deacetylases, enhance histone acetylation and chromatin accessibility, and thereby increase transcriptional accessibility at relevant gene regulatory regions. Some microbial metabolites may also influence one-carbon metabolism and indirectly affect DNA methylation [30,31]. However, direct experimental evidence in piglets showing that specific early colonizers shape long-term immune developmental trajectories through DNA methylation or histone acetylation remains limited [11]. Further validation through multi-omics and functional experiments in piglet models is needed. In addition to epigenetic regulation, microbiota-derived metabolites can influence long-term intestinal resilience by shaping mucosal immune development. SCFAs, especially acetate, propionate, and butyrate, are not only energy substrates for epithelial cells but also immunoregulatory signals. They can participate in the regulation of Treg differentiation, IL-10 production, IL-22-mediated epithelial repair, antimicrobial peptide expression, and inflammatory threshold setting. If early colonization is disrupted, reduced production of these metabolites may weaken immune tolerance and barrier-associated defense during later weaning stress. Therefore, early microbial metabolite exposure may contribute to immune imprinting by influencing how the mucosal immune system responds to commensals, pathogens, and dietary transition.

Thus, the formation of the intestinal host–microbiota interaction network in piglets is a dynamic process jointly driven by maternal seeding, niche filtering, nutritional substrate availability, host counter-selection, and early developmental programming. As this network is gradually established, the relationship between the gut microbiota and host is no longer simply one of colonization and host accommodation. Instead, it involves continuous bidirectional regulation through metabolites, immune factors, barrier-related molecules, and neuroendocrine signals [12,30].

## 4. Molecular Mechanisms of Host–Microbiota Interactions

Host–microbiota interactions are not governed by a single signaling pathway but instead arise from the coordinated actions of microbial metabolites, host-derived secretory factors, mucosal immune responses, and gut–brain axis signaling [32]. Microbial metabolites serve as key mediators that translate changes in microbial community composition into host-perceived signals affecting intestinal barrier function, immune regulation, and metabolic homeostasis. Conversely, host-derived secretions and immune molecules can shape the composition, activity, and spatial organization of the gut microbiota [25,33]. Some associations have been validated in piglet studies, including the beneficial effects of SCFAs on intestinal barrier integrity and the relationship between probiotic supplementation and the alleviation of post-weaning diarrhea. In contrast, a substantial proportion of evidence regarding the AHR/IL-22 axis, HIF-1α signaling, miRNA-containing extracellular vesicles, and several gut–brain axis mechanisms is still derived from rodent models, human studies, or in vitro systems and therefore requires further validation in piglets [32,34,35,36,37,38].

### 4.1. Signaling Roles of Microbial Metabolites

**Figure 1:**
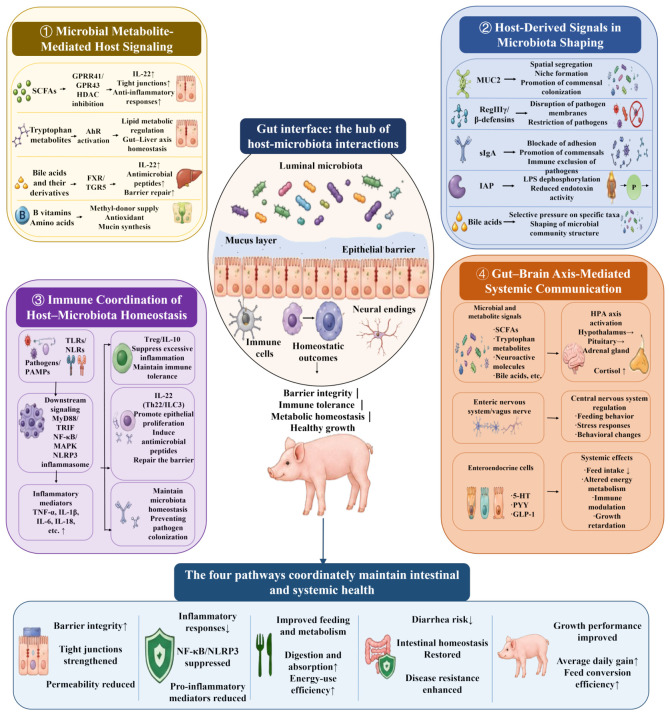
Four major molecular axes by which bidirectional host–microbiota interactions regulate gut health in piglets. ① Microbial metabolite-mediated host signaling. SCFAs, tryptophan metabolites, bile acid derivatives, B vitamins, amino acids, and other microbial metabolites regulate epithelial barrier function, immune responses, lipid metabolism, gut–liver homeostasis, antioxidant defense, and mucin synthesis through pathways such as GPR41/GPR43, HDAC inhibition, AhR, and FXR/TGR5 signaling. ② Host-derived signals in microbiota shaping. MUC2, antimicrobial peptides, sIgA, IAP, and bile acids shape microbial composition and spatial distribution by maintaining the mucus barrier, restricting pathogen expansion, promoting selected commensals, reducing LPS activity, and exerting selective pressure on specific microbial taxa. ③ Immune coordination of host–microbiota homeostasis. Microbial signals and PAMPs are recognized by TLRs and NLRs, activating MyD88/TRIF, NF-κB/MAPK, and NLRP3-related inflammatory pathways. In parallel, Treg/IL-10 and IL-22-producing Th22/ILC3 responses limit excessive inflammation, maintain immune tolerance, induce antimicrobial peptides, and support epithelial repair. ④ Gut–brain axis-mediated systemic communication. Microbial metabolites and neuroactive molecules interact with the enteric nervous system, vagus nerve, enteroendocrine cells, and HPA axis to regulate feeding behavior, stress responses, immune modulation, energy metabolism, and growth performance. Together, these pathways strengthen barrier integrity, suppress inflammation, improve digestion and energy-use efficiency, reduce diarrhea risk, restore intestinal homeostasis, enhance disease resistance, and support healthy growth. SCFAs: Short-chain fatty acids; HDAC: Histone deacetylase; AhR: Aryl hydrocarbon receptor; FXR: Farnesoid X receptor; TGR5: Takeda G protein-coupled receptor 5; MUC2: Mucin 2; sIgA: Secretory immunoglobulin A; IAP: Intestinal alkaline phosphatase; LPS: Lipopolysaccharide; PAMPs: Pathogen-associated molecular patterns; TLRs: Toll-like receptors; NLRs: NOD-like receptors; MyD88: Myeloid differentiation primary response 88; TRIF: TIR-domain-containing adapter-inducing interferon-β; NF-κB: Nuclear factor kappa B; MAPK: Mitogen-activated protein kinase; NLRP3: NOD-like receptor family pyrin domain-containing 3; Treg: Regulatory T cell; ILC3: Group 3 innate lymphoid cell; HPA axis: Hypothalamus–pituitary–adrenal axis.

Microbial metabolites are key molecular mediators through which the microbiota affects the host. The piglet gut microbiota can utilize substrates such as milk oligosaccharides, plant polysaccharides, proteins, and amino acids to produce SCFAs, branched-chain fatty acids, indole metabolites, secondary bile acids, polyamines, vitamins, and neuroactive substances [39,40]. These metabolites enter signaling networks in epithelial cells, immune cells, and endocrine cells, thereby modulating barrier repair, inflammatory responses, energy utilization, and neural regulation (Figure 1). Compared with changes in microbial composition, metabolite profiles more directly represent the functional outputs of the gut microbiota [6,12]. However, alterations in metabolite abundance do not necessarily indicate causal effects. Establishing causality requires further evidence from intervention studies, receptor-targeting approaches, or strain-specific validation experiments.

#### 4.1.1. SCFAs

SCFAs are representative microbiota-derived signaling molecules generated mainly through the fermentation of dietary fiber, resistant starch, and selected oligosaccharides. Acetate, propionate, and butyrate are the major SCFAs in the intestine. Acetate can serve as an energy substrate for peripheral tissues and participate in lipid metabolism. Propionate contributes to hepatic gluconeogenesis and immune regulation, whereas butyrate is a primary energy source for colonocytes. In weaned piglets, SCFAs help maintain intestinal homeostasis by supporting epithelial energy supply, barrier repair, and mucosal immune regulation. Reduced feed intake, insufficient fermentable substrates, and fluctuations in acid-producing bacteria after weaning may collectively decrease SCFA production and weaken these protective functions.

Beyond their nutritional role, SCFAs act as signaling molecules by activating G protein-coupled receptors such as GPR41 and GPR43 and by inhibiting histone deacetylases. These pathways can promote IL-22 production by CD4+ T cells and innate lymphoid cells and enhance epithelial protection through aryl hydrocarbon receptor (AhR) and hypoxia-inducible factor 1-α (HIF-1α)-related signaling. IL-22 further induces antimicrobial peptide expression, supports epithelial repair, and reinforces barrier integrity. Thus, microbial fermentation products are not merely metabolic end products; they also mediate the conversion of dietary changes into mucosal immune and epithelial responses [30,31]. Recent studies have further expanded the biological significance of SCFAs beyond epithelial energy supply and classical immune regulation. One important direction is the regulation of enteroendocrine and neuroendocrine signaling [41]. One emerging direction is their involvement in enteroendocrine and neuroendocrine signaling. SCFAs can act on enterochromaffin cells and regulate intestinal serotonin, also known as 5-hydroxytryptamine (5-HT), which contributes to intestinal motility, secretion, epithelial communication, and gut–brain signaling. Through enterochromaffin cells, serotonin-related pathways, vagal communication, and the enteric nervous system, SCFAs may connect microbial fermentation activity with feeding behavior, intestinal transit, stress responses, and post-weaning adaptation [42]. This mechanism is especially relevant in weaned piglets because reduced feed intake, altered fermentation substrate supply, and microbial dysbiosis may jointly change SCFA production and neuroendocrine signaling during the early post-weaning period. Another emerging area is the effect of SCFAs on intestinal epithelial renewal and intestinal stem cell activity. The intestinal epithelium is continuously regenerated by stem and progenitor cells located in crypt regions. After weaning, villus atrophy, crypt remodeling, barrier dysfunction, and inflammatory stimulation increase the demand for epithelial repair. SCFAs, particularly butyrate and propionate, may influence epithelial renewal by regulating the balance between stem-cell proliferation, differentiation into absorptive or secretory epithelial lineages, mitochondrial metabolism, and Wnt-related signaling [43]. These effects may help explain why SCFA-producing bacteria and fermentable substrates are associated with improved intestinal morphology and barrier recovery. However, direct evidence in piglets remains less complete than evidence from organoid, rodent, or human epithelial models. Therefore, the SCFA–intestinal stem cell axis should be presented as a promising mechanism that requires further validation in piglet intestinal organoids and in vivo weaning models.

In piglet models, experimental studies have provided support for the protective effects of SCFAs on intestinal barrier function. These effects are mainly reflected in enhanced intestinal epithelial energy supply, increased tight-junction protein expression, improved intestinal morphology, and alleviation of inflammatory responses [44]. SCFA infusion can increase acetate, propionate, butyrate, and total volatile fatty acid levels in serum and intestinal contents. It can also enhance SCFA receptor expression, reduce epithelial-cell apoptosis, upregulate Occludin and Claudin-1, and decrease inflammatory cytokine expression. Studies on *Bacillus velezensis* MZ09 further extend the SCFA mechanism to GPR43/STAT3 signaling, IL-10 production, mitochondrial homeostasis, and NLRP3 inflammasome-mediated pyroptosis [27,30,31,44,45]. SCFAs also have systemic metabolic effects that extend beyond the intestinal mucosa. Acetate, propionate, and butyrate can enter the portal circulation and interact with hepatic metabolism through the gut–liver axis. Propionate may participate in gluconeogenic regulation, acetate can contribute to lipid-related metabolic pathways, and butyrate may influence energy homeostasis and inflammatory tone. In pig studies, SCFA supplementation has been linked with changes in lipid and glucose metabolism and liver metabolite profiles, indicating that microbial fermentation products may connect intestinal microbial activity with systemic metabolic adaptation [46]. This perspective is important for weaned piglets because post-weaning stress is accompanied not only by diarrhea and barrier injury but also by reduced feed intake, altered energy utilization, and growth retardation. The strength of evidence differs among these SCFA-related mechanisms. In piglets, relatively direct evidence supports the effects of SCFAs on intestinal morphology, tight-junction expression, inflammatory responses, and systemic glucose and lipid metabolism. By contrast, their regulation of enterochromaffin cell-derived serotonin, direct enteric nervous system activation, and intestinal stem cell proliferation or differentiation has been characterized more extensively in non-porcine models or organoid systems. These mechanisms are biologically relevant to piglet intestinal health, but further piglet-specific validation is needed before they can be regarded as established causal pathways in post-weaning diarrhea or growth recovery [47].

#### 4.1.2. Tryptophan Metabolites

Tryptophan metabolites are another important class of microbe-derived signals. Commensal bacteria, including certain lactic acid bacteria, can convert tryptophan into indole and its derivatives. These metabolites can act as AhR ligands; activate the AhR/IL-22 axis; and promote epithelial repair, antimicrobial peptide expression, and mucosal immune balance. Tryptophan can also enter the kynurenine and serotonin pathways, thereby linking immune regulation, metabolic homeostasis, and neuroendocrine responses [11].

Weaning stress may jointly alter the direction of tryptophan metabolism through changes in nutrient substrate availability, microbial metabolic function, and inflammatory signaling. On the one hand, reduced feed intake decreases the intake of amino acids such as tryptophan and the availability of luminal substrates. On the other hand, microbial dysbiosis may reduce the capacity to produce indole and its derivatives, weakening AhR-mediated protective signals. Meanwhile, under inflammatory conditions, the kynurenine pathway may be further activated, shifting tryptophan metabolism away from barrier protection and immune homeostasis toward an inflammation-associated metabolic profile [6,48]. This may render the intestinal barrier more prone to low-grade inflammation and increased permeability. Studies involving *Lactococcus lactis* have demonstrated that specific lactic acid bacterial interventions can influence host serum tryptophan concentrations, sulfur-containing amino acid metabolism in the intestinal mucosa, and the gut gamma-aminobutyric acid (GABA) ergic signaling system. These findings suggest that lactic acid bacteria may contribute to the maintenance of intestinal homeostasis in weaned piglets through the modulation of amino acid metabolism and neuroactive signaling pathways. However, much of the mechanistic evidence supporting the tryptophan-AHR/IL-22 axis is still derived from rodent models, human studies of inflammatory bowel disease, or in vitro experiments. Therefore, further validation in piglet models is required, particularly with respect to the roles of specific bacterial strains, defined microbial metabolites, and host receptor-mediated signaling pathways [48].

#### 4.1.3. Bile Acid Transformation

Bile acid transformation reflects the bidirectional metabolic relationship between the host and microbiota. The host synthesizes primary bile acids in the liver and secretes them into the intestine. The gut microbiota generates various secondary bile acids through deconjugation, dehydroxylation, and related reactions. These bile acid derivatives act on receptors such as FXR and TGR5 to regulate lipid absorption, energy metabolism, inflammatory responses, and barrier function [49].

In piglets, bile acid metabolism is not only an important component of fat digestion, absorption, and lipid homeostasis, but may also participate in intestinal absorption, hepatic transport, and inflammatory regulation of certain feed-derived toxic compounds through the gut–liver axis. Research on bile acid metabolism has expanded studies of host–microbiota interactions beyond the intestinal barrier and mucosal immunity to broader pathophysiological processes such as feed-contaminant transport, gut–liver axis signaling, and the regulation of hepatic injury. However, this mechanism is likely to be toxin-specific. Recent evidence from piglet models suggests that microbiota-mediated bile acid transformation may contribute to the intestinal absorption and enterohepatic transport of aflatoxin B1 (AFB1) [49]. This process may be associated with reduced bile salt hydrolase (BSH) activity in the ileal microbiota, subsequent alterations in the bile acid pool, and disruption of bile acid-related gut–liver signaling. However, comparable in vivo evidence in piglets remains insufficient for deoxynivalenol (DON) and zearalenone (ZEN), and the AFB1-related mechanism should not be directly extrapolated to these mycotoxins. For DON, most bile acid-related findings are derived from human intestinal epithelial in vitro models, indicating potential effects on epithelial barrier integrity, microbial metabolic activity, and the expression of bile acid transport-associated molecules. For ZEN, available evidence mainly comes from mouse models or in vitro studies using human gut microbiota, suggesting that ZEN-induced toxicity may involve gut microbiota remodeling, altered bile acid metabolism, and modulation of FXR signaling, thereby affecting its enterohepatic circulation and enteric–hepatic toxicity. Collectively, the interaction between bile acid metabolism and feed-borne mycotoxins appears to be highly toxin-specific and host species-dependent. Further mechanistic validation in distinct mycotoxin exposure models, particularly piglet models, is still required [49].

#### 4.1.4. Vitamin and Amino Acid Metabolites

In addition to the metabolism of SCFAs and bile acids, vitamin- and amino acid-related metabolic processes also play important roles in maintaining intestinal homeostasis in piglets. Methionine, for example, is not only an essential amino acid required for protein synthesis but also participates in S-adenosylmethionine production, antioxidant responses, maintenance of immune function, and protection of the epithelial barrier. Lee (2025) found that methionine deficiency affects the expression of tight junction-related markers and enhances cell death-related signaling [50]. After methionine supplementation, methionine metabolism, pyruvate metabolism, and several immune- and barrier-related pathways were upregulated [29,51].

Gut microorganisms participate in the synthesis of B vitamins and in the production and transformation of various amino acid metabolites. Their capacity to synthesize and transform these metabolites is often highly species- and strain-specific. A multi-omics study of weaned piglets found that weaning stress reduced the relative abundance of commensal *Lactobacillus*, with particularly marked decreases in *L. mucosae*, *L. reuteri*, and *L. amylovorus* [52]. Meanwhile, intestinal levels of metabolites related to *L-methionine*, serine, glycine, threonine, and cysteine, as well as the branched-chain amino acids isoleucine and valine, also tended to decline. Subsequent in vitro culture experiments further showed that *Lactobacillus* isolates from weaned piglets could produce methionine, serine, glycine, threonine, branched-chain amino acids, and compounds related to homocysteine and putrescine. These findings suggest that the loss of *Lactobacillus* during weaning reflects not only microbial compositional changes but also a decline in microbial support for amino acid production and transformation. In addition to lactic acid bacteria, other gut microorganisms also possess varying capacities for amino acid synthesis and conversion. Genome reconstruction studies have shown that many *Bifidobacterium* strains harbor the genetic basis for synthesizing multiple amino acids. In members of Bacteroidetes, such as *Bacteroides* and *Prevotella*, alternative enzyme systems related to arginine synthesis have been detected. For example, *Bacteroides fragilis* can participate in arginine biosynthesis through succinylated intermediates.

Amino acid metabolism is not only part of host nutrient supply but also an important functional component of host–microbe interactions. After weaning stress, the reduction in protective gut microorganisms may decrease the supply of amino acids and related amino acid metabolites, thereby further weakening intestinal epithelial repair, antioxidant defense, and immune homeostasis. At the same time, supplementation with specific amino acids may improve the intestinal microbial ecosystem by selectively promoting the growth and metabolic activity of commensal bacteria [53]. The recently proposed concept of an “amino acid-mediated antibiotic-like effect” suggests that functional amino acids such as methionine, arginine, and threonine not only serve as basic substrates for protein synthesis and nutrient metabolism but also indirectly regulate microbial ecology by modifying the intestinal nutritional microenvironment, promoting beneficial bacterial colonization, inhibiting the proliferation of potential pathogens, enhancing mucus barrier formation, and modulating mucosal immune responses [29]. Dietary crude protein level is another important factor shaping amino acid availability and microbial metabolism in the gut. When dietary crude protein is excessive or poorly digestible, more undigested protein and endogenous nitrogen enter the distal intestine, providing substrates for proteolytic fermentation. This process can promote the production of ammonia, biogenic amines, branched-chain fatty acids, phenolic compounds, and indole-related metabolites. Although some amino acid-derived metabolites have physiological signaling functions, excessive protein fermentation may increase luminal pH, epithelial irritation, inflammatory stimulation, and diarrhea risk. Therefore, the effect of dietary protein on piglet intestinal health should be interpreted not only from the perspective of growth requirement but also from the perspective of host–microbiota metabolic balance.

### 4.2. Shaping of the Microbiota by Host Signals

Microorganisms can affect the host, and the host can reciprocally shape the microbiota. The intestine actively participates in selecting colonizing microorganisms. Intestinal epithelial cells, goblet cells, Paneth cells, and mucosal immune cells continuously produce host-derived factors such as mucins, antimicrobial peptides, sIgA, bile acids, and intestinal alkaline phosphatase. Together, these molecules participate in establishing and maintaining the intestinal microecological boundary by regulating the spatial distance between microbes and the epithelial surface, limiting the access of potential pathogens to the mucosal layer, and providing or restricting niches for specific commensals (Figure 1). They thereby influence microbial composition, spatial distribution, and functional status.

#### 4.2.1. Intestinal Alkaline Phosphatase and Lipopolysaccharide (LPS)-Mediated Inflammatory Regulation

Intestinal alkaline phosphatase (IAP) is secreted by intestinal epithelial cells and is one of the important host molecules that limits pro-inflammatory microbial stimulation in the intestinal lumen. IAP can reduce the immunostimulatory activity of microbe-associated molecular patterns (MAMPs) such as LPS through dephosphorylation, thereby alleviating excessive activation of TLR4-related inflammatory pathways. IAP also helps maintain tight-junction integrity, regulate spatial separation at the microbe–epithelium interface, and reduce translocation of bacteria and bacterial components across the intestinal epithelial barrier [54,55].

In weaned piglets, the protective role of IAP is mainly reflected in its regulation of inflammatory stimulation derived from Gram-negative bacteria. Post-weaning microbial dysbiosis may promote the expansion of Gram-negative bacteria such as *E. coli*, increase LPS exposure, and enhance TLR4-mediated inflammatory responses. Under these conditions, higher IAP activity can reduce the pro-inflammatory effects of LPS through dephosphorylation, thereby alleviating excessive activation of mucosal immunity and helping maintain intestinal barrier homeostasis. It should be noted that the role of IAP in the piglet intestine still requires further validation through stage-specific and intestinal segment-specific studies. Existing evidence more strongly supports IAP as an important regulatory node through which the host limits microbe-derived pro-inflammatory stimulation and maintains homeostasis at the lumen–mucosa interface, rather than as a mature intervention target supported by sufficient experimental evidence. Future studies should therefore clarify changes in IAP expression; enzymatic activity; and its causal relationships with LPS load, barrier function, and microbial spatial distribution across different intestinal segments before and after weaning [54,56].

#### 4.2.2. Mucins and Antimicrobial Peptides Maintain Microbial Boundaries

The mucus layer forms the first physical and biochemical interface between the host and gut microbiota. Mucins such as MUC2, secreted by goblet cells, cover the intestinal epithelial surface and form a mucus structure with barrier and spatial-separation functions. This structure reduces direct contact between bacteria and epithelial cells and helps maintain an appropriate distance between commensal communities and host tissues, thereby contributing to the maintenance of the intestinal microecological boundary and mucosal homeostasis [9]. The mucus layer not only spatially separates microorganisms from epithelial cells but also participates in intestinal niche selection. Some commensals can use mucin glycans as nutritional substrates and thereby gain a colonization advantage near the mucus layer. This substrate selectivity also affects competitive relationships and spatial distribution among different microbial taxa. Weaning stress weakens goblet cell function, reduces mucin secretion, and disrupts tight-junction integrity, thereby diminishing the spatial-separation capacity of the intestinal barrier and allowing potential pathogens to approach the intestinal epithelium more readily [10,12].

Antimicrobial peptides participate in active host selection of the gut microbiota by selectively suppressing potential pathogens and regulating microbial spatial distribution. Molecules such as RegIIIγ and β-defensins, secreted by Paneth cells and intestinal epithelial cells, can limit the expansion of potential pathogens and maintain spatial separation between microorganisms and host tissues. In weaned piglets, reduced MUC2 expression, impaired tight-junction integrity, and dysregulated antimicrobial peptide expression all weaken the spatial separation between luminal microorganisms and the epithelial surface. Under these conditions, potential pathogens such as Enterotoxigenic *Escherichia coli* (ETEC) are more likely to breach the mucus barrier and approach intestinal epithelial cells, increasing the risk of adhesion, colonization, inflammatory activation, and aggravated barrier injury [10].

#### 4.2.3. Host miRNA Exosomes and Cross-Kingdom Regulation

Recent studies have shown that host-derived miRNAs can enter the intestinal lumen through carriers such as exosomes and participate in interspecies communication between the host and gut microorganisms [57]. This phenomenon provides a new mechanistic explanation for how the host actively regulates microbial community composition and microbial functional status. Intestinal epithelial cells release miRNA-containing exosomes into the intestinal lumen, where these miRNAs can be taken up by specific bacteria and influence bacterial gene expression, growth, and metabolic activity [58].

Liu (2016) found that mouse and human feces are enriched with host-derived miRNAs, which are mainly produced by intestinal epithelial cells and Hopx-positive cells [58]. Impaired miRNA biogenesis in intestinal epithelial cells leads to marked changes in the composition of the mouse gut microbiota, whereas supplementation with wild-type fecal miRNAs can partially restore the disrupted microbial community structure. These findings suggest that fecal miRNAs are not merely residual nucleic acids derived from epithelial cell shedding or the release of cellular debris but may function as host-derived nucleic acid signals involved in regulating the intestinal microbial ecosystem [45].

Some miRNAs can be taken up by gut bacteria and affect bacterial gene transcription and growth activity. Host cells can directly participate in shaping the functional state of the microbiota through miRNA-mediated cross-kingdom information transfer. In vitro culture experiments have shown that different host-derived miRNAs exert distinct regulatory effects on gut bacteria [25]. miR-515-5p can promote the proliferation of *Fusobacterium nucleatum* and increase its 16S rRNA transcript abundance, whereas miR-1226-5p can promote the growth of *Escherichia coli* and upregulate its *yegH* mRNA expression. Related studies also found that miR-4747-3p can increase RNase P-related transcript levels in *E. coli*, whereas miR-1224-5p and miR-663 can reduce the mRNA expression of *rutA* and *fucO*. These studies suggest that the effects of host-derived miRNAs on gut bacteria show a certain degree of gene targeting and functional specificity. Their effects are reflected not only in changes in bacterial proliferation but may also involve regulation of the transcriptional expression of specific bacterial genes, thereby altering bacterial metabolic capacity and functional behavior and contributing to active host regulation of gut microbial community structure and function.

This mechanism has also been preliminarily validated in models of intestinal injury and diarrhea in weaned piglets. In a model of post-weaning stress-induced diarrhea in piglets, Zhou (2022) found significant changes in fecal microbiota composition, metabolite profiles, and miRNA expression levels in diarrheic piglets [59]. Mechanistic studies showed that decreased expression of ssc-miR-425-5p and ssc-miR-423-3p weakened their negative regulation of the fumarate reductase gene (*frd*) in *Prevotella*. This enhanced succinate production-related metabolic activity in this bacterial group, leading to abnormal accumulation of microbially derived succinate in the colon. Succinate accumulation promoted transepithelial chloride secretion by epithelial cells and activated macrophage inflammatory responses through MyD88-dependent TLR4 signaling, thereby aggravating diarrhea.

Research on host miRNA-mediated cross-kingdom regulation in piglets is still at an early stage. Existing evidence is mainly derived from a small number of weaning diarrhea models and specific miRNA molecules. A complete causal chain is still lacking to determine whether epithelial-derived miRNAs can enter the intestinal lumen through exosomes; specifically regulate bacterial functional genes; and further affect microbial metabolism, barrier integrity, and mucosal inflammatory responses. Future studies should systematically evaluate the universality and practical value of host-derived miRNAs in regulating the intestinal microbial ecosystem of piglets across different ages, intestinal segments, and stress backgrounds.

#### 4.2.4. Effects of Host Genetics on the Microbiome

In recent years, the influence of host genetic background on gut microbial composition, colonization patterns, and functional outputs has become an active research topic. mGWAS studies link host genotype with microbial abundance features, enabling more systematic analysis of the relationships among host genetic background, the gut microbiome, and host phenotypes. Host genetics may affect microbial structure and production phenotypes by altering epithelial niches, immune thresholds, and the nutritional environment [60]. In a study of host–microbiota interactions in pigs, Wang (2025) found that *Bacteroidales*_RF16_group may be associated with host genetic regulatory signals, with candidate genes including Epidermal Growth Factor, Glutamyl Aminopeptidase, and Caspase 6 [61,62,63]. These genes do not directly determine microbial composition but may alter intestinal barrier status and mucosal niche conditions by regulating epithelial renewal, protein or peptide metabolism, apoptosis, and local immune responses, thereby influencing changes in the abundance of *Bacteroidales*_RF16_group [26].

Studies of associations between host genetics and the gut microbiota provide a new theoretical basis and research direction for precision breeding and individualized microecological intervention [64]. Host genotype may participate in shaping microbial composition and variation in intervention responses by affecting microbial colonization, mucosal immunity, and the intestinal microenvironment. However, existing mGWAS results mostly indicate correlations or potential regulatory signals and are insufficient to prove that specific candidate genes alone determine intestinal health phenotypes in piglets [65,66,67].

### 4.3. Immune Regulation of Host–Microbiota Balance

The mucosal immune system plays an important regulatory role in host–microbiota interactions by coordinating immune tolerance toward commensals and defensive responses against pathogens, thereby maintaining the homeostasis of the intestinal microecosystem and barrier function [4]. At the intestinal mucosal interface, sIgA, antimicrobial peptides, and mucus-associated molecules together form an important defense line that limits microbial access to the epithelium (Figure 1). These immune and barrier factors not only help inhibit pathogen adhesion, colonization, and invasion but also maintain an appropriate spatial distance between commensals and epithelial cells, forming a dynamic balance between microbial defense and commensal tolerance [68,69]. During the weaning period, the mucosal immune system of piglets remains incompletely developed, resulting in a limited capacity to appropriately regulate responses to microbial stimuli. Under the combined influence of nutritional, environmental, and pathogen-related stressors, immune interactions between the host and the gut microbiota are more likely to deviate from homeostasis, leading to excessive amplification of inflammatory signaling and dysregulation of immune responses [36,70,71].

Pattern-recognition receptors provide an important molecular basis for host sensing of intestinal microorganisms and their structural components. By recognizing microbe-associated molecular patterns, they activate downstream signaling pathways and participate in regulating mucosal immune responses and inflammatory levels [72]. Intestinal epithelial cells and immune cells recognize microbial molecules such as LPS, peptidoglycan, flagellin, and bacterial DNA through receptors including Toll-like receptors and NOD-like receptors [73,74]. Moderate microbial signaling promotes maturation of the mucosal immune system and development of the intestinal barrier, whereas excessive or persistent stimulation activates pathways such as NF-κB, MAPK, and inflammasomes, inducing the expression and release of pro-inflammatory cytokines or chemokines such as TNF-α, IL-1β, IL-6, and IL-8 [37].

Commensal communities train the host immune system during development. Through microbe-associated molecular patterns and metabolites, they continuously act on the intestinal mucosal immune system, promoting immune-cell differentiation, establishment of immune tolerance, and maturation of barrier-associated defenses, thereby improving the host capacity to regulate pathogen invasion and inflammatory stimulation. Studies have shown that the immune system can read microbial metabolite signals and convert these signals into epithelial protective responses [75,76]. sIgA, Treg cells, IL-10, and SCFAs jointly participate in maintaining intestinal mucosal immune homeostasis. Among these factors, sIgA mainly restricts bacterial adhesion and regulates microbial spatial distribution, Treg cells and IL-10 suppress excessive inflammatory responses, and SCFAs enhance immune tolerance by promoting Treg differentiation and regulating immune-cell function. Notably, IL-22 constitutes a key node connecting microbial metabolism, mucosal immunity, and epithelial repair. SCFAs can promote IL-22 production by CD4+ T cells and innate lymphoid cells, and IL-22 then acts on intestinal epithelial cells to induce antimicrobial peptide expression and promote barrier repair [2,10,77].

Weaning stress disrupts the dynamic balance among the microbiota, immunity, and barrier function. When barrier permeability increases, microbe-associated molecules such as LPS, peptidoglycan, and flagellin can more readily enter the mucosal layer and aberrantly activate pathways such as TLR4/MyD88/NF-κB, MAPK, and the NLRP3 inflammasome. This increases the release of pro-inflammatory factors and further aggravates damage to tight junctions and the mucus layer [27,78,79]. Therefore, intestinal injury during weaning should be understood as the result of reciprocal amplification among microbial dysbiosis, mucosal immune imbalance, and impaired barrier function, rather than as a process that can be fully explained by a single pathogenic stimulus.

### 4.4. Neuroendocrine Communication in the Gut–Brain Axis

Changes in the gut microbiota can also influence systemic physiological responses through the gut–brain axis. Bidirectional communication among microorganisms, the intestine, and the central nervous system mainly depends on immune, endocrine, and neural signaling pathways. Intestinal inflammatory factors can regulate neuroendocrine activity, and activation of the hypothalamic–pituitary–adrenal (HPA) axis further affects intestinal motility, secretory function, and barrier permeability. Meanwhile, the vagus nerve and enteric nervous system participate in transmitting microbial metabolic signals to the central nervous system, thereby linking local intestinal microecological changes with systemic stress responses (Figure 1).

Piglet weaning involves not only nutritional changes but also marked psychological and physiological stress. Maternal separation, environmental change, transport, mixing, and social regrouping all affect the stress state of piglets. Within a short period after weaning, piglets may show body-weight loss, increased salivary cortisol, altered colonic microbial composition, and changes in SCFAs, indicating that weaning stress can simultaneously alter neuroendocrine status, microbial structure, and metabolite profiles [80]. The gut microbiota can produce or regulate various neuroactive substances, including GABA, 5-hydroxytryptamine (5-HT), dopamine, norepinephrine, and their precursors. Microbial metabolites such as SCFAs can participate in host stress regulation through enterochromaffin cells, the enteric nervous system, and the vagus nerve. Tryptophan metabolism is a key node in the microbiota–gut–brain axis. It provides substrates for the synthesis of neuroactive substances such as 5-HT and can also generate indole AhR ligands through microbial metabolism, thereby linking neuroendocrine regulation, mucosal immunity, and barrier repair [40,81,82].

Production-related stress can disrupt microbial homeostasis and increase intestinal permeability, promoting the entry of microbe-associated molecules such as LPS into the mucosal layer and inducing inflammatory responses. Inflammatory signals can further affect the Hypothalamic–pituitary–adrenal axis and systemic metabolic status, causing local intestinal disturbance to extend into systemic phenotypes such as reduced feed intake and growth retardation. The gut–brain axis provides an additional perspective for understanding the pathogenesis of post-weaning diarrhea. However, current evidence remains insufficient to clearly establish the causal relationships and temporal sequence among stress responses, microbial dysbiosis, and pathogen infection. Further studies integrating longitudinal sampling, multi-omics approaches, and targeted intervention experiments are needed to clarify these interactions and validate the underlying mechanisms [39,40].

## 5. Imbalance of the Interaction Network Under Weaning Stress

**Figure 2:**
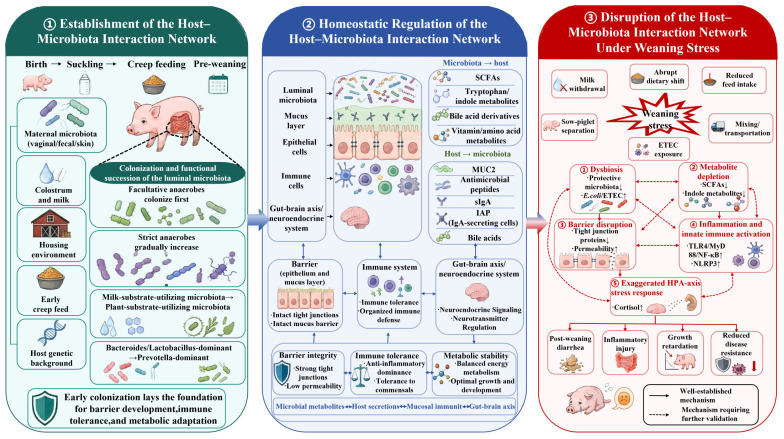
Establishment, homeostatic regulation, and weaning stress-induced disruption of the host–microbiota interaction network in piglets. ① Establishment of the host–microbiota interaction network. From birth to pre-weaning, maternal microbiota, colostrum and milk, the housing environment, early creep feed, and host genetic background jointly shape microbial colonization and functional succession in the piglet intestine. Facultative anaerobes colonize first and promote the expansion of strict anaerobes, while the microbiota gradually shifts from milk-substrate-utilizing communities to plant-substrate-utilizing communities. This early colonization process supports barrier development, immune tolerance, and metabolic adaptation. ② Homeostatic regulation of the host–microbiota interaction network. Under stable conditions, luminal microbiota, the mucus layer, epithelial cells, immune cells, and the gut–brain/neuroendocrine system interact bidirectionally. Microbial metabolites, including SCFAs, tryptophan/indole metabolites, bile acid derivatives, and vitamin- or amino acid-related metabolites, regulate barrier integrity, immune balance, and metabolic stability. Host-derived factors such as MUC2, antimicrobial peptides, sIgA, IAP, IgA-secreting cells, and bile acids further shape microbial spatial organization and community structure, thereby maintaining low permeability, immune tolerance, and normal growth. ③ Disruption of the host–microbiota interaction network under weaning stress. Milk withdrawal, abrupt dietary shift, reduced feed intake, sow–piglet separation, mixing or transportation, and ETEC exposure can disturb microbial ecology and host adaptation. These stressors induce dysbiosis, depletion of protective metabolites, epithelial barrier damage, inflammatory activation, and exaggerated HPA-axis responses, ultimately contributing to post-weaning diarrhea, intestinal injury, growth retardation, and reduced disease resistance. Solid arrows indicate relatively well-established mechanisms, whereas dashed arrows indicate mechanisms requiring further validation. SCFAs: Short-chain fatty acids; MUC2: Mucin 2; sIgA: Secretory immunoglobulin A; IAP: Intestinal alkaline phosphatase; ETEC: Enterotoxigenic *Escherichia coli*; HPA axis: Hypothalamus–pituitary–adrenal axis.

Weaning is a critical window during which the piglet host–microbiota interaction network is particularly susceptible to destabilization. At this time, intestinal structure and function are not yet fully mature. Digestive enzyme secretion, villus and crypt architecture, tight junctions, the mucus layer, and mucosal immunity remain in developmental and adaptive stages. A marked mismatch therefore develops between rapid changes in external stimuli and insufficient host regulatory capacity, making weaning a typical perturbation model for studying host–microbiota interaction imbalance. When the weaning transition, referring to the combined shift from sow milk-based suckling to independent solid-feed intake together with maternal separation and environmental change, is gradual and well managed, piglets can progressively complete microbial functional remodeling and host adaptation. In contrast, when weaning is abrupt or accompanied by additional stressors, such as ETEC infection, low feed intake, temperature stress, and high stocking density, the host–microbiota interaction network is more likely to become imbalanced, driving adverse outcomes such as diarrhea, inflammation, and impaired growth (Figure 2).

After weaning, the intestinal microbial ecology of piglets undergoes rapid remodeling, commonly characterized by decreases in protective lactic acid bacteria and expansion of facultative anaerobes and potential pathogens. Importantly, this post-weaning remodeling is not limited to taxonomic changes but also involves functional reorganization in substrate utilization, fermentation capacity, redox balance, and inflammatory potential. Under diarrheal or stress conditions, the abundance of beneficial bacteria such as *Limosilactobacillus mucosae*, *Limosilactobacillus reuteri*, and *Lactobacillus amylovorus* decreases, whereas opportunistic pathogens such as *E. coli* and functions related to LPS biosynthesis increase. Reduced feed intake further decreases the availability of fermentable substrates, while dietary transition from milk-derived nutrients to plant-based solid feed reshapes the luminal nutrient environment (Figure 2). As a result, microbial dysbiosis during weaning should be understood not only as a compositional disturbance but also as a reduction in beneficial metabolic outputs and an increase in pro-inflammatory microbial pressure [39].

ETEC infection is an important amplifier of post-weaning host–microbiota interaction imbalance. ETEC F4 or F18 can exploit ecological niches created by microbial dysbiosis during weaning and adhere to and colonize the small intestinal epithelium through fimbrial binding to epithelial receptors. Its heat-labile enterotoxin promotes chloride secretion and inhibits sodium absorption through the cyclic adenosine monophosphate (cAMP) pathway, whereas heat-stable enterotoxins disrupt ion transport and paracellular permeability through cyclic guanosine monophosphate or calcium signaling pathways. Among them, heat-stable enterotoxin b (STb) may also downregulate tight-junction proteins such as Claudin-1, ZO-1, and Occludin. At the same time, ETEC infection further aggravates microbial dysbiosis, weakens protective fermentation functions, and reduces volatile fatty acid production, thereby promoting progression of diarrhea and barrier injury. Thus, ETEC converts microbial ecological imbalance into epithelial secretory dysfunction, barrier disruption, and inflammatory amplification. Importantly, ETEC infection does not act independently of the resident microbiota. Instead, it may create a self-reinforcing cycle in which microbial dysbiosis facilitates pathogen expansion, pathogen-derived toxins and microbe-associated molecular patterns damage the epithelial barrier, barrier injury increases microbial translocation and immune activation, and inflammation further reshapes the intestinal microbial ecosystem [77,83].

## 6. Omics Technologies for Deciphering Host–Microbiota Interactions

Host–microbiota interactions exhibit marked multilayered and cross-scale regulation, involving microbial structure, metabolic function, the epithelial barrier, mucosal immunity, neuroendocrine signaling, and other processes. Single indicators are no longer sufficient to explain the mechanisms underlying post-weaning diarrhea and barrier injury. It is therefore necessary to integrate analyses across multiple levels, including microbial composition, functional genes, metabolite profiles, host cellular responses, and the activation status of key signaling pathways. Multi-omics technologies have consequently become important tools for deciphering host–microbiota interaction networks in piglets. Recent pig studies using integrated omics approaches provide more concrete examples of how microbial changes are linked with host molecular responses during weaning. These studies show that multi-omics integration can move beyond the description of microbial community shifts and identify candidate microbe–metabolite–host gene axes related to barrier dysfunction, immune activation, metabolic adaptation, and inflammatory regulation [84]. However, most of these studies remain association-based. Therefore, the regulatory axes identified by multi-omics should be interpreted as mechanistic clues or putative causal pathways that require further validation through targeted intervention, metabolite rescue, receptor blockade, or functional experiments in piglet models.

### 6.1. Microbiome Sequencing: From Taxonomic Composition to Functional Potential

16S rRNA sequencing and metagenomic sequencing are mainly used to analyze intestinal microbial structure and functional changes during weaning. 16S rRNA sequencing is suitable for assessing microbial composition, changes in dominant taxa, and alpha and beta diversity. It is relatively low cost and suitable for large-sample screening. However, because 16S rRNA sequencing is primarily taxonomic and has limited resolution for functional inference, it cannot fully explain how microbial changes are translated into metabolic or host-response alterations. Metagenomic sequencing provides higher resolution and can identify not only microbial composition but also functional genes, metabolic pathways, and potential virulence factors. For studies of intestinal imbalance during weaning, describing changes in genus abundance alone is insufficient. Greater attention should be given not only to taxonomic composition but also to microbial functional potential. Key microbial functions, including carbohydrate degradation, amino acid metabolism, LPS synthesis, bile acid transformation, and short-chain fatty acid production, should be evaluated to better understand how microbial communities contribute to intestinal homeostasis or dysbiosis in weaned piglets [15,22].

### 6.2. Metabolomics: Linking Microbial Remodeling with Functional Outputs

Metabolomics can be used to analyze changes in intestinal metabolite profiles and is an important means of determining whether changes in microbial structure are translated into functional metabolic outputs and host physiological effects. SCFAs, bile acids, amino acid metabolites, lipid metabolites, and vitamin-related metabolites can all serve as mediators linking microbial changes with host barrier, immune, and metabolic phenotypes. Studies in weaned piglets have shown that weaning not only alters gut microbial composition but also remodels amino acid metabolism, lipid metabolism, energy metabolism, and bile acid-related pathways. Compared with microbial analysis alone, metabolomics is better able to reveal the functional connections among microbial changes, barrier gene expression, and systemic metabolic responses [39,80]. A representative integrated-omics study in commercial piglets combined cecal microbiota profiling, jejunal host gene-expression analysis, and serum metabolomics around weaning. This study showed that weaning was accompanied by rapid microbial remodeling; downregulation of genes related to tight junctions, mucin production, and nutrient transport; and marked systemic metabolic changes, including altered lipid, amino acid, and ketone-body metabolism. The integrated analysis suggested that microbiota shifts after weaning are coordinated with impaired epithelial barrier and digestive functions as well as systemic metabolic stress. This case supports the concept that microbial dysbiosis during weaning should not be interpreted only as a taxonomic disturbance, but as part of a broader host–microbiota–metabolite response network.

### 6.3. Host-Side Omics: Transcriptomics, Proteomics, and Single-Cell Resolution

Single-cell transcriptomics can resolve host responses induced by weaning stress at cellular resolution. Compared with conventional bulk tissue transcriptomics, which reflects only overall gene-expression changes, single-cell RNA sequencing can further distinguish cell-type composition, functional states, and intercellular communication among epithelial cells, goblet cells, immune cells, stromal cells, and other cell populations. Single-cell atlases of the ileal mucosa in weaned piglets indicate that T-cell subsets, T helper 17 cells (Th17) functional states, and cytotoxic T-cell features are remodeled after weaning, allowing inflammatory responses to be localized to specific cell types and molecular states. This technology is therefore more suitable than measurement of individual inflammatory factors for revealing cellular mechanisms of mucosal immune imbalance during weaning [3].

Transcriptomics, proteomics, and pathway analysis can reveal key host-side response mechanisms under weaning stress. Metagenomics and metabolomics mainly reflect changes in the microbiota and its metabolic outputs, whereas whether the host mounts corresponding functional responses must be further verified through gene expression, protein abundance, and signaling pathway activation. After weaning, processes such as intestinal barrier function, nutrient transport, inflammatory responses, oxidative stress, mitochondrial function, and cell death may all be remodeled. Integrating these host responses with microbial functions and metabolite changes can provide clearer evidence for elucidating the mechanistic links between microbial metabolites and host signaling pathways. Although multi-omics approaches have improved our understanding of host–microbiota interaction networks, their main strength still lies in identifying potential associations. Metagenomics, metabolomics, transcriptomics, proteomics, and single-cell transcriptomics can help screen microbial taxa, functional genes, metabolites, host cellular responses, and signaling pathways associated with intestinal barrier injury, immune activation, metabolic changes, and diarrhea-related phenotypes. However, these findings usually reflect coordinated changes across different biological layers. They do not, on their own, prove that a specific strain, metabolite, or pathway directly causes a given host phenotype. Therefore, multi-omics results should be regarded as a basis for further mechanistic studies rather than as definitive evidence of causality. To clarify the functional roles of these candidate factors, key strains need to be isolated through culturomics and further evaluated using microbial or metabolite interventions, organoid or organ-on-a-chip co-culture models, and targeted animal experiments. In this context, culturomics and organ-on-a-chip technologies serve as important bridging tools that link omics-based discovery with the development of mechanism-based nutritional intervention strategies.

### 6.4. Organ-on-a-Chip Platforms for Functional Validation of Host–Microbiota Interactions

Organ-on-a-chip and multi-organ-on-a-chip platforms should not be viewed only as advanced in vitro models. They can also be used to validate candidate mechanisms identified by multi-omics studies and to support the design of mechanism-based nutritional intervention strategies. In studies of piglet intestinal health, multi-omics analyses can identify candidate strains, microbial metabolites, host receptors, inflammatory pathways, and barrier-related targets associated with post-weaning dysbiosis, barrier injury, and intestinal dysfunction [39,85]. These candidate factors can then be further tested in organ-on-a-chip systems to determine whether they exert direct biological effects under conditions that partially resemble the intestinal microenvironment. Compared with conventional two-dimensional cell culture systems or relatively static organoid models, microphysiological chip systems can better reproduce fluid shear stress, epithelial barrier interfaces, oxygen gradients, mechanical stimulation, and the dynamic exchange of nutrients and microbial metabolites. This is particularly relevant to studies of host–microbiota interactions. The production of microbial metabolites, changes in epithelial permeability, activation of immune signaling, and communication between the gut and the liver or brain are continuous biological processes. They cannot be fully captured by measurements taken at a single time point. Previous reviews have suggested that organoids and organ-on-a-chip technologies can help reconstruct key aspects of host–microbe interactions in vitro. This may reduce the uncertainty associated with mechanistic inferences based only on animal experiments or metagenomic association data [84,86]. In addition, recent advances in multi-organ-on-a-chip systems have shown further potential, especially through fluidically coupled organ modules, integrated sensors, automated perfusion, and real-time monitoring. These features make such systems useful for studying inter-organ communication and biological responses induced by drugs or metabolites [87].

In piglet intestinal health research, these platforms are better viewed as tools for mechanistic validation rather than as replacements for live piglet models. Candidate strains, microbial metabolites, and host signaling pathways identified through metagenomics, metabolomics, transcriptomics, or single-cell analyses can first be functionally tested in porcine intestinal epithelial organoids or gut-on-a-chip systems. Readouts such as transepithelial electrical resistance, tight junction protein expression, mucus secretion, inflammatory cytokine release, epithelial renewal, and metabolite transport can help determine whether a specific microbiota–metabolite–host pathway has a direct biological effect. Looking further ahead, gut-on-a-chip–liver axis, gut–immune axis, or gut–brain axis models may provide more controllable platforms for evaluating the systemic effects of microbial metabolites, including bile acid derivatives, SCFAs, tryptophan metabolites, and amino acid-derived signals [86]. However, the application of these technologies in piglet research still faces several technical limitations. Stable co-culture of intestinal epithelial cells, anaerobic microorganisms, immune cells, and organ-specific cellular components requires precise control of oxygen tension, medium composition, flow rate, and microbial overgrowth. Therefore, organ-on-a-chip models should be regarded as complementary mechanistic validation systems that connect multi-omics analysis with animal experiments, rather than as independent evidence sufficient to explain post-weaning diarrhea symptoms or changes in production performance.

More importantly, these platforms can serve as a mechanistic screening step before nutritional strategies are tested in large-scale piglet trials. For example, potential probiotics isolated from the piglet intestine, postbiotic components, SCFAs, tryptophan metabolites, bile acid derivatives, amino acid-derived signaling molecules, and plant-derived bioactive compounds identified through multi-omics approaches can first be evaluated in gut-on-a-chip systems. These models allow researchers to examine whether such factors affect key processes such as epithelial permeability, mucus secretion, inflammatory signaling, metabolite transport, and epithelial repair. This validation step can help determine whether a nutritional intervention directly targets biologically relevant host–microbiota pathways. It may also reduce the uncertainty associated with advancing intervention strategies based only on correlative omics evidence.

### 6.5. Culturomics for Functional Validation of Candidate Bacteria

Culturomics is an important step in moving microbiota association studies toward functional validation. Sequencing technologies can identify potential functional bacteria, but without culturable live strains, it is difficult to conduct colonization validation, host–microbe or microbe-microbe co-culture assays, metabolite detection, or safety evaluation. By optimizing culture media, oxygen conditions, temperature, incubation time, and screening strategies, culturomics can improve the efficiency of isolating intestinal bacteria from piglets and provide an experimental basis for verifying whether candidate bacteria possess functions such as SCFA production, ETEC antagonism, tight-junction protection, and immune-tolerance regulation [3,29].

At present, porcine gut culturomics is still less mature than human gut microbiome research in terms of strain library size, standardized culture conditions, genome-level annotation, and the public availability of well-characterized isolates. Nevertheless, this approach is of particular value for studies of porcine intestinal health. Many gut bacteria are adapted to specific host environments and may show species-specific patterns in colonization ability, substrate utilization, and interactions with the piglet mucosa. Therefore, sequence-based prediction alone is often insufficient to determine whether these bacteria have practical application potential. Compared with metagenomic or metabolomic analyses, the isolation of live porcine intestinal strains allows more direct evaluation of their growth characteristics, metabolite-producing capacity, epithelial adhesion, anti-pathogen activity, antimicrobial resistance risk, and safety-related features. This is particularly important when metagenomic or metabolomic data identify potentially beneficial microbial taxa or functional pathways but cannot determine whether the corresponding live bacteria can be developed into stable probiotic candidates.

Therefore, culturomics can serve as a practical bridge between multi-omics discovery and targeted microbiota-based nutritional interventions. Candidate strains can be isolated from healthy piglets, sows, or piglets with stronger adaptation after weaning. Particular attention should be given to microorganisms that may be associated with higher SCFAs production, stronger barrier-protective effects, reduced inflammatory signaling, or resistance to enterotoxigenic *Escherichia coli*. These strains can then be systematically evaluated through genome sequencing, metabolite profiling, epithelial cell co-culture assays, and organ-on-a-chip models. Through this workflow, culturomics can help convert microbial features identified by omics analyses from descriptive biomarkers into experimentally testable functional strains, probiotic candidates, postbiotic sources, or defined microbial consortia. This provides a more concrete basis for improving intestinal health in weaned piglets.

### 6.6. Multi-Omics Integration and Causal Validation

The core value of multi-omics integration lies in linking microbial changes, metabolite outputs, host cellular responses, and signaling pathway activation to construct “microbiota–metabolite–host pathway” interaction networks. Metagenomics addresses how the microbiota and its functional potential change. Metabolomics reveals changes in metabolite profiles and functional metabolic outputs. Single-cell transcriptomics identifies specific responding cell populations and cell-state transitions. Transcriptomics and pathway analyses reflect the activation status of host signaling. It should be noted that multi-omics networks mainly reveal covariation and cannot, by themselves, prove that a specific strain or metabolite directly causes a host response. Relevant causal chains still require further validation through strain isolation, colonization experiments, metabolite interventions, and microbiota reconstruction models [55,88].

A more appropriate research strategy is to proceed from multi-omics association screening toward mechanistic validation. Candidate microbial groups and functional genes can first be screened through metagenomics; key metabolites can then be identified by metabolomics; host-responding cells and signaling pathways can be resolved using transcriptomics or single-cell transcriptomics; live strains can subsequently be obtained through culturomics; and causal relationships can finally be validated through strain supplementation, metabolite intervention, receptor blockade, organoid co-culture, or simplified microbiota colonization experiments. This stepwise strategy helps transform descriptive associations into testable mechanistic hypotheses and ultimately into causally supported intervention targets for improving piglet intestinal health.

In summary, multi-omics, culturomics, and organ-on-a-chip platforms can be integrated into a closed-loop research framework for studies of piglet intestinal health. Multi-omics approaches are mainly used for discovery. They help identify key microbial taxa, functional genes, metabolites, host-responsive cell populations, and signaling pathways associated with post-weaning intestinal dysfunction. Culturomics further provides live bacterial resources for strain-level characterization, functional screening, safety assessment, and the development of probiotic candidates or defined microbial consortia. In contrast, organ-on-a-chip platforms and related ex vivo systems are more suitable for mechanistic validation. These models can help determine whether candidate strains, metabolites, or nutritional components directly affect epithelial barrier integrity, mucosal immune responses, microbial metabolite transport, or inter-organ communication. Through this workflow, phenotypic associations, mechanistic hypothesis generation, functional validation, and intervention development can be more closely connected. This provides a more reliable methodological basis for developing microbiota-targeted nutritional strategies for weaned piglets.

## 7. Microbiota-Based Strategies for Improving Piglet Intestinal Health

In the context of restricted antibiotic use, strategies for improving piglet intestinal health should move beyond a linear “single-additive” model. Greater attention should be given to the multilevel restoration of host–microbiota interaction networks. Post-weaning diarrhea is rarely caused by a single factor. It usually results from several interconnected disturbances, including microbial dysbiosis, reduced production of protective metabolites, impaired epithelial barrier repair, excessive activation of mucosal immune responses, and limited adaptation to dietary transition and environmental stress. Therefore, nutritional interventions should not be evaluated only according to additive type, such as probiotics, prebiotics, postbiotics, organic acids, or plant-derived extracts. A more useful framework is to classify these strategies according to their main site of action and the functional disturbance they are intended to correct.

**Figure 3:**
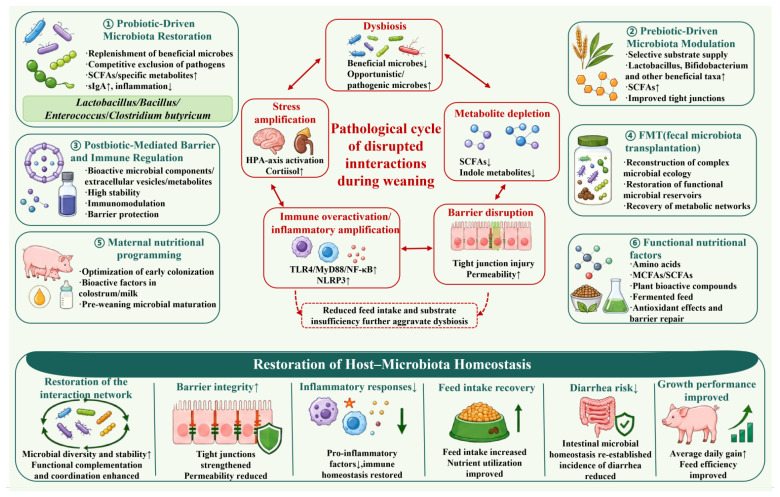
Mechanistic framework of microbiota-targeted strategies for directed repair of disrupted host–microbiota interactions during weaning. ① Probiotic-driven microbiota restoration. Probiotics such as *Lactobacillus*, *Bacillus*, *Enterococcus*, and *Clostridium butyricum* help replenish beneficial microbes, exclude pathogens, increase SCFA-related metabolites, enhance sIgA production, and reduce inflammation. ② Prebiotic-driven microbiota modulation. Prebiotics selectively promote beneficial taxa, including *Lactobacillus* and *Bifidobacterium*, thereby increasing SCFA production and supporting tight-junction integrity. ③ Postbiotic-mediated barrier and immune regulation. Postbiotics, including microbial components, extracellular vesicles, and metabolites, provide stable bioactive signals that support immunomodulation and barrier protection. ④ Fecal microbiota transplantation. FMT helps reconstruct complex microbial communities, restore functional microbial reservoirs, and recover metabolic networks. ⑤ Maternal nutritional programming. Maternal nutritional intervention promotes early colonization and pre-weaning microbial maturation through bioactive factors in colostrum and milk. ⑥ Functional nutritional factors. Amino acids, MCFAs, SCFAs, plant bioactive compounds, and fermented feed enhance antioxidant defense, barrier repair, microbial stability, and metabolic recovery. Together, these strategies restore host–microbiota homeostasis, improve barrier integrity and feed utilization, suppress inflammation, reduce diarrhea risk, and promote growth performance in weaned piglets. SCFAs: Short-chain fatty acids; MCFAs: Medium-chain fatty acids; sIgA: Secretory immunoglobulin A; FMT: Fecal microbiota transplantation; HPA axis: Hypothalamus–pituitary–adrenal axis; TLR4: Toll-like receptor 4; MyD88: Myeloid differentiation primary response 88; NF-κB: Nuclear factor kappa B; NLRP3: NOD-like receptor family pyrin domain-containing 3.

Based on this rationale, microbiota-targeted nutritional strategies can be broadly divided into four interconnected levels. The first level is microbial community remodeling, which mainly includes probiotics and FMT. These approaches aim to replenish beneficial bacteria, limit pathogen expansion, and help restore a disturbed microbial ecosystem. The second level is modulation of the metabolic microenvironment. This includes prebiotics, postbiotics, SCFAs, medium-chain fatty acids (MCFAs), organic acids, and fermented feed. These interventions mainly improve luminal substrate availability, metabolite composition, and microbial fermentation patterns. The third level is support for host barrier and immune function. This category includes functional amino acids, plant-derived extracts, and traditional Chinese medicine formulations, which are mainly related to epithelial repair, mucus secretion, antioxidant defense, and inflammatory regulation. The fourth level is systemic nutritional management, including low-protein diets and maternal nutritional interventions. These strategies do not target a single bacterium or pathway. Instead, they act by adjusting the overall dietary structure or early-life developmental environment, reducing harmful fermentation pressure, improving adaptation to feed transition, and shaping the early establishment of host–microbiota interactions. These four levels are not separate from one another. Rather, they work together to support the restoration of intestinal homeostasis in weaned piglets (Figure 3).

### 7.1. Rationale for Hierarchical Restoration of Network Dysbiosis

Intestinal health in weaned piglets is not determined by a single factor. Instead, it is jointly influenced by microbial community structure, microbial metabolites, epithelial barrier status, mucosal immune responses, and overall nutritional adaptability. These factors are closely connected. Disruption in one component may further amplify dysfunction in others. For example, reduced feed intake or an abrupt change in diet after weaning can directly alter the availability of intestinal substrates. This may change microbial fermentation patterns, reduce the production of protective metabolites, and impair epithelial repair while promoting inflammatory responses.

Therefore, when evaluating nutritional interventions, attention should not be limited to whether a single additive produces a specific effect. It is also important to determine which key processes are mainly improved by the intervention. For example, an intervention may help stabilize the microbial ecosystem, optimize the intestinal metabolic environment, strengthen barrier and immune function, or improve the ability of piglets to adapt during the weaning transition. This analytical approach can reduce the overly simple interpretation of “one factor–one effect”. It also better reflects the multifactorial interactions involved in the regulation of piglet intestinal health.

### 7.2. Microbial Community Remodeling

The first stage of network restoration focuses mainly on microbial community remodeling. Its core aim is to restore the abundance and niche functions of beneficial bacteria, enhance intestinal colonization resistance, limit the expansion of potential pathogens, and gradually re-establish the microbial ecological balance disrupted by weaning stress.

#### 7.2.1. Probiotics: Strain-Specific Microbial Supplementation

Probiotics are commonly used microecological tools in studies of antibiotic replacement for weaned piglets. Lactobacilli, Bifidobacteria, Bacilli, and *Clostridium butyricum* have all received considerable attention. Their mechanisms of action mainly include competitive exclusion of pathogens, production of antimicrobial metabolites, enhancement of tight junctions, promotion of sIgA secretion, suppression of pro-inflammatory responses, and optimization of microbial structure, thereby improving intestinal barrier function and reducing the risk of post-weaning intestinal injury [89,90,91].

Meta-analyses support an overall protective effect of probiotics on the intestinal barrier of weaned piglets, mainly reflected in decreased diarrhea incidence, increased ZO-1 and Occludin expression, and improved jejunal villus height. However, the effects of probiotic interventions are influenced by strain type, dosage, route of administration, trial duration, weaning age, dietary background, and sampled intestinal segment, resulting in heterogeneity among studies. Practical application should therefore emphasize strain specificity and context adaptation and avoid overgeneralizing probiotic effects across different production or experimental settings [92].

Strain specificity is a central issue in probiotic research and application. Different strains do not have identical effects on immune regulation, barrier repair, and microbial remodeling. *Lactobacillus reuteri* derived from Ningxiang pigs can increase IgA, IgG, and sIgA levels; improve ileal villus structure; and upregulate ZO-1 and Claudin-1 expression (Figure 3). *Lactobacillus rhamnosus* GG can improve the mucus layer, tight junctions, and immune indicators under rotavirus challenge. *Bifidobacterium animalis* JYBR-190 can improve growth, diarrhea, and some aspects of microbial structure, but its regulation of tight junctions and inflammatory cytokines may not occur synchronously [82,93,94]. These findings indicate that improved production performance, microbial optimization, and repair of barrier molecules do not always occur simultaneously and that probiotic efficacy should be evaluated based on strain specificity and an integrated assessment of growth performance, diarrhea outcomes, microbial ecology, barrier integrity, and immune status [88].

Bacillus-based preparations have relatively high processing stability and application convenience in the feed industry because of their heat resistance, storage stability, and pelleting stability compared with lactic acid bacteria. *Bacillus subtilis*, *Bacillus pumilus*, and their combinations have been reported to improve growth performance and reduce diarrhea incidence in weaned piglets, while regulating inflammation- and tight-junction-related indicators. Mechanistic studies of *Bacillus velezensis* MZ09 suggest that its protective effects may be associated with increased SCFAs, activation of GPR43/STAT3 signaling, upregulation of IL-10, and inhibition of the NLRP3 inflammasome [88,93,94].

#### 7.2.2. FMT: Ecosystem-Level Microbial Reconstruction

FMT differs from single-probiotic intervention. Its core purpose is not to supplement a limited number of functional strains, but to transfer a relatively complete microbial ecosystem, together with its associated metabolic capacity, from a healthy donor to reconstruct the intestinal microecology of the recipient [46,93].

In recent years, FMT studies in weaned piglets have moved beyond phenotypic indicators such as diarrhea incidence and growth performance to focus on how FMT regulates host–microbe interface processes, including microbial reconstruction, barrier repair, mucosal immunity, and restoration of metabolic function. Studies have shown that FMT can reshape microbial structure at the small intestinal level in weaned piglets, accompanied by changes in metabolic function and host gene expression (Figure 3). FMT can enrich beneficial genera related to Bifidobacteriaceae, regulate carbohydrate, amino acid, nucleotide, and vitamin metabolism and affect expression of genes related to immunity, barrier function, and neuroendocrine regulation [46,69]. These results suggest that FMT effects go beyond fecal microbial changes and may participate in maintaining host–microbe interface homeostasis by reshaping local small intestinal ecology, regulating host transcriptional responses, and enhancing resistance to pathogens [93].

Industrial application of FMT still faces many practical limitations, including donor screening, exclusion of pathogenic microorganisms and antimicrobial resistance genes, batch stability, standardization of dosing, and optimization of intervention timing. Oral gavage provides stronger controllability but is more costly and labor-intensive. Feed inclusion is more convenient for production applications, but it is difficult to ensure microbial viability and colonization after feed processing and gastrointestinal passage. Therefore, FMT is currently more suitable as a tool for mechanistic analysis and functional-bacterium screening than as a routine large-scale production strategy. In research, FMT can first be used to verify the protective effects of healthy microbial networks. Culturomics and multi-omics technologies can then be combined to screen key strains or metabolites, ultimately transforming them into probiotic, postbiotic, or metabolite preparations with defined components, lower safety risks, and controllable quality.

### 7.3. Metabolic Microenvironment Modulation

The second level of network restoration mainly focuses on the regulation of the metabolic microenvironment. During weaning, reduced feed intake, abrupt changes in diet composition, and gut microbial imbalance may all weaken the production of beneficial fermentation products. They may also promote the conversion of undigested nutrients into harmful metabolites. The main goal of interventions at this level is to optimize luminal substrate availability, promote the production of protective microbial metabolites such as SCFAs, limit proteolytic fermentation and other unfavorable metabolic processes, and provide a more suitable local environment for epithelial repair and mucosal immune homeostasis.

#### 7.3.1. Prebiotics: Substrate-Based Regulation of Microbial Metabolism

Prebiotics are substrate-based microecological interventions. They are selectively utilized by beneficial bacteria in the host intestine, thereby promoting a more favorable microbial structure, enhancing the production of beneficial metabolites, and improving host intestinal health. Common prebiotics include Fructo-oligosaccharides (FOS), Galacto-oligosaccharides (GOS), Xylo-oligosaccharides (XOS), Mannan-oligosaccharides (MOS), and inulin. Prebiotics can selectively promote the proliferation of protective bacteria such as lactobacilli and bifidobacteria; suppress the expansion of potential pathogens such as *Escherichia coli* and *Escherichia Shigella*; and increase SCFA levels, including acetate, propionate, and butyrate [81]. These effects collectively contribute to improvements in the intestinal microecology, barrier function, and mucosal immune homeostasis of piglets (Figure 3).

Studies have shown that inulin supplementation can improve small intestinal villus morphology and ZO-1 distribution, reduce serum Diamine oxidase (DAO) and intestinal mucosal TNF-α levels, and increase cecal acetate and butyrate concentrations, suggesting that it may exert prebiotic effects by enhancing beneficial bacterial fermentation, promoting SCFA production, and repairing the epithelial barrier. In ETEC challenge models, MOS also show substantial intestinal protective potential. MOS can increase IgA and IgM levels; reduce pro-inflammatory cytokine expression; improve villus morphology and digestive enzyme activity; and upregulate ZO-1, Claudin-1, and nutrient-transport-related molecules. Its protective effects may involve multiple mechanisms, including competition with pathogen adhesion, immune enhancement, inflammation alleviation, improved digestion and absorption, and strengthening of tight junctions [95,96,97].

Prebiotic application should not be simply equated with increasing dietary fiber levels. Because digestive function in piglets is not fully mature, excessive or unsuitable fermentable substrates may increase the flow of undigested nutrients into the hindgut and promote expansion of potentially unfavorable microbial groups. Prebiotic effects are influenced by substrate type, fermentability, particle size, compatibility with exogenous enzymes, and dietary background. Therefore, prebiotics should be regarded as precision substrate-regulation tools rather than simply as fiber additives [4].

#### 7.3.2. Postbiotics: Stable Microbe-Derived Bioactive Preparations

Postbiotics are microecological interventions based on inactivated microbial cells and their structural components, including inactivated cells, cell-wall components, fermentation products, and microbe-derived active substances. The ISAPP consensus emphasizes that postbiotics should have defined health effects and well-characterized components (Figure 3). Therefore, not all dead bacteria, bacterial fragments, or simple metabolites should be generalized as postbiotics [91,98].

The advantages of postbiotics lie in their stability and safety. Postbiotics do not require live-cell colonization to exert effects and can reduce potential risks associated with live microbial preparations, including carriage of antimicrobial resistance genes, abnormal proliferation, and ecological safety concerns. Extracellular vesicles derived from lactic acid bacteria provide new evidence for postbiotic development [99]. Studies have found that extracellular vesicles from *Limosilactobacillus mucosae* can alleviate diarrhea-like symptoms induced by ETEC K88 and participate in inflammation control by regulating macrophage phenotypes, suggesting that nonviable microbial structures or secreted components can also mediate some probiotic protective effects [100,101,102].

The development and application of postbiotics still face challenges. Because microbial sources, preparation processes, and component compositions differ substantially among products, the key active material basis, mechanisms of action, batch stability, immunogenicity, and dosing standards require further clarification and standardization. For piglet production, postbiotics should be developed as microbe-derived active preparations with defined components, verifiable effects, and controllable quality, rather than being broadly generalized as all fermentation products or inactivated bacterial preparations.

#### 7.3.3. SCFAs, MCFAs, Organic Acids, and Fermented Feed: Direct Modulation of the Luminal Metabolic Environment

SCFAs, mainly including acetate, propionate, and butyrate, are important functional metabolites produced by microbial carbohydrate fermentation. In weaned piglets, SCFAs are closely associated with intestinal energy supply, epithelial barrier integrity, immune regulation, and microbial homeostasis. Among them, butyrate is particularly important. It serves as a major energy source for intestinal epithelial cells and also helps regulate tight junction maintenance and inflammatory signaling. Experimental studies have shown that intragastric infusion of SCFAs in piglets can increase SCFA concentrations in serum and intestinal digesta. It can also enhance the expression of SCFA receptors, such as GPR41 and GPR43, and upregulate tight junction-related genes, including Occludin and Claudin-1. In addition, SCFA infusion has been reported to increase the abundance of lactobacilli while reducing intestinal *Escherichia coli* abundance and inflammatory marker levels. These findings suggest that SCFAs may alleviate weaning-related intestinal injury through several mechanisms. These include energy provision, barrier repair, microbial regulation, and maintenance of immune homeostasis. However, the efficacy of exogenous SCFA supplementation is influenced by dose, release site, palatability, absorption rate, and the fermentability of the basal diet. Therefore, coated butyrate products, SCFA salts, fermentable fibers, and prebiotic substrates should be considered as part of an integrated strategy rather than being evaluated as isolated interventions.

MCFAs, mainly including caproic, caprylic, capric, and lauric acids, have different functional characteristics from SCFAs. Compared with SCFAs, MCFAs are more closely related to antimicrobial activity and the regulation of energy metabolism. Their antimicrobial effects are generally linked to disruption of bacterial lipid membranes, altered membrane permeability, and reduced pathogen survival. Monoglyceride forms, such as glycerol monolaurate, may show stronger biological activity than free fatty acids because of their greater membrane-targeting ability and favorable physicochemical stability. Studies in weaned piglets have shown that dietary MCFA supplementation can reduce diarrhea incidence, improve antioxidant status, decrease the production of inflammatory cytokines, and modulate gut microbiota composition. Recent evidence also suggests that combined supplementation with MCFAs and SCFAs may serve as a partial alternative to high-dose zinc oxide. This strategy may improve growth performance; alleviate oxidative stress and inflammatory responses; and increase the abundance of beneficial bacteria, such as lactobacilli and *Roseburia*. These findings indicate that functional fatty acids have potential in zinc oxide reduction and antibiotic-alternative strategies. However, their practical efficacy still depends on fatty acid chain length, esterified or coated forms, inclusion level, and the dietary matrix.

Organic acids are important nutritional components for regulating the luminal environment in weaned piglets. Commonly used organic acids include formic acid, fumaric acid, lactic acid, citric acid, propionic acid, benzoic acid, and butyric acid. One of their main functions is to reduce the acid-binding capacity of feed and regulate gastrointestinal pH. This can promote pepsin activation, protein hydrolysis, and digestive adaptation during the early post-weaning period. Some organic acids can also enter bacterial cells in their undissociated form. They may disrupt intracellular pH homeostasis and thereby exert bacteriostatic or bactericidal effects.

In weaned piglets, supplementation with blended organic acids has been reported to improve average daily gain and feed conversion efficiency. It may also increase immunoglobulin levels; improve the villus height-to-crypt depth ratio; and upregulate the expression of tight junction-related genes, such as Claudin-1 and ZO-1. In addition, organic acids may increase the concentrations of volatile fatty acids in the cecum and colon. These findings suggest that the effects of organic acids are not limited to acidification alone. They may also involve improved nutrient digestion, modulation of microbial fermentation, and enhancement of epithelial barrier function. However, their efficacy is closely related to acid type, dose, combination, and release site. Excessive acidification or inappropriate acid combinations may reduce feed palatability or limit delivery to the target intestinal segment. Therefore, dose–response relationships, coated or sustained-release forms, and administration methods should be carefully optimized in practical applications.

Functional oligosaccharides can also be included in the regulation of the metabolic microenvironment, mainly as substrate-oriented modulators. XOS, arabinoxylan, and MOS do not act primarily through direct acidification. However, they can influence microbial fermentation, pathogen adhesion, oxidative stress, and immune responses. In weaned piglets, supplementation with XOS or arabinoxylan has been reported to reduce diarrhea incidence, increase intestinal antioxidant enzyme activity, elevate sIgA and IL-10 levels, promote the growth of lactobacilli and bifidobacteria, and enhance intestinal organic acid production. Selenium-enriched MOS has also been shown to alleviate ETEC-induced diarrhea by reducing oxidative stress and inflammation, improving mucosal barrier function, and modulating the gut microbiota. Therefore, these compounds are better regarded as fermentable substrates or adhesion-blocking functional carbohydrates. They may work together with SCFAs, MCFAs, organic acids, and fermented feed to support the restoration of the luminal metabolic environment.

Overall, SCFAs, MCFAs, organic acids, functional oligosaccharides, and fermented feed should not be viewed simply as stand-alone replacements for antibiotics or zinc oxide. Their greater value lies in their combined ability to improve the unfavorable luminal conditions that occur after weaning, including pathogen expansion, increased inflammation, and disordered fermentation patterns. By regulating substrate supply, acidification status, microbial metabolites, and barrier-supporting functions, these interventions may help shift the intestinal environment toward a more stable state that is more favorable for epithelial repair. Future studies should further examine their efficacy under production conditions, the optimal ratios of functional fatty acids, coating and release characteristics, interactions among dietary additives, and response patterns during different post-weaning windows. Multi-omics approaches may also help determine whether changes in microbial composition are accompanied by functional changes in organic acid production, epithelial energy metabolism, and immune tolerance.

### 7.4. Host Barrier and Immune Restoration

The third level of network restoration mainly focuses on the recovery of host barrier function and mucosal immunity. When gut microbial dysbiosis and metabolic disturbances persist, epithelial repair capacity, mucus barrier maintenance, antioxidant defense, and the regulation of inflammatory thresholds often become key determinants of whether intestinal homeostasis can be re-established. Therefore, functional amino acids, plant-derived extracts, and traditional Chinese medicine formulations should not be regarded merely as conventional feed additives. Instead, they can be considered host-directed modulators that enhance epithelial barrier resilience, improve antioxidant status, and maintain mucosal immune homeostasis.

#### 7.4.1. Functional Amino Acids: Support for Barrier Repair, Mucus Synthesis, and Immune Homeostasis

Amino acids are not only substrates for protein synthesis but also important functional nutritional factors that regulate the intestinal barrier, mucus synthesis, antioxidant defense, and mucosal immunity. Glutamine, arginine, threonine, methionine, cysteine, and tryptophan can indirectly reduce the risk of pathogen expansion by supporting barrier repair, regulating microbial structure, and altering the microbial metabolic environment. The proposal of amino acid-mediated antibiotic-like effects has extended amino acid research beyond traditional evaluation of nutrient requirements toward functional precision nutritional intervention [80].

#### 7.4.2. Phytogenic Extracts and Traditional Chinese Medicine Formulas: Multi-Target Regulation of Inflammation and Barrier Integrity

Plant extracts and traditional Chinese medicine compound formulas have multi-component, multi-target, and multi-pathway regulatory characteristics. They can improve piglet intestinal health through antioxidant, anti-inflammatory, barrier-repair, microbial-regulatory, and mucosal immune-enhancing pathways. Existing studies suggest that some plant bioactive compounds and traditional Chinese medicine compound formulas have the potential to alleviate barrier injury, improve microbial metabolism, and reduce diarrhea risk under conditions of mycotoxin exposure, inflammation, or weaning stress [4,55,82]. However, because of their complex composition, further work is needed to strengthen component standardization, identification of active substances, and validation of mechanistic chains.

### 7.5. Systemic Nutritional Management Strategies

The fourth level of network restoration mainly involves systemic nutritional management rather than the direct supplementation of a single functional additive. Low-protein diets and maternal nutritional regulation can modulate host–microbiota interactions at a broader regulatory level. They do so by altering nutrient supply, microbial substrate availability, early microbial colonization trajectories, and the developmental status of the intestinal barrier and mucosal immune system. The importance of these strategies lies in the fact that they do not only target established microbial imbalance. They also address upstream factors that contribute to dysbiosis, including excessive nitrogen fermentation, insufficient adaptation to feed transition during weaning, and unstable microbial succession in early life.

#### 7.5.1. Low-Protein Diets: Regulation of Nitrogen Flow and Microbial Fermentation Pressure

Crude protein reduction is an important but often underestimated systemic nutritional strategy for improving host–microbiota interactions in weaned piglets. After weaning, digestive function in piglets is not yet fully stable. When dietary crude protein levels are too high, more undigested proteins, peptides, amino acids, and endogenous nitrogen-containing substrates may reach the hindgut. These substrates can promote proteolytic fermentation and increase the production of metabolites such as ammonia, biogenic amines, branched-chain fatty acids, phenolic compounds, and some indole derivatives. When these products accumulate excessively, they may disturb luminal pH, irritate the epithelium, impair barrier function, and promote mucosal inflammation, thereby increasing the risk of diarrhea.

From a microbial ecological perspective, the effect of low-protein diets is not limited to reducing nitrogen intake. More importantly, they reshape microbial substrate availability and fermentation patterns in the hindgut. When less undigested protein enters the distal intestine, the substrate pressure that supports the expansion of proteolytic bacteria is reduced. If an adequate supply of fermentable carbohydrates is also provided, saccharolytic bacteria and short-chain fatty acid-producing microbes may become more functionally important. Previous studies have shown that dietary protein level can alter the relative abundance of fermentation-related taxa, including *Prevotella*, *Coprococcus*, *Streptococcus*, Peptostreptococcaceae-related bacteria, *Roseburia*, Lachnospiraceae, and Ruminococcaceae. However, these taxa should not be simply classified as “beneficial” or “harmful”. For example, *Prevotella* is functionally heterogeneous. Some members participate in polysaccharide degradation and carbohydrate fermentation, whereas others may utilize peptides or amino acids under specific dietary conditions. Therefore, when evaluating low-protein diets, greater attention should be paid to the overall balance between proteolytic and saccharolytic fermentation rather than to changes in a single genus.

Low-protein diets may also affect microbial nitrogen metabolism. A reduced flow of protein substrates into the hindgut may suppress bacterial amino acid deamination, decarboxylation, and aromatic amino acid fermentation. This can reduce the production of ammonia, amines, branched-chain fatty acids, phenolic compounds, and some indole metabolites. It should be noted that these metabolites do not all have uniformly negative effects. For instance, some tryptophan-derived indole derivatives can activate aryl hydrocarbon receptor signaling and support epithelial defense. However, excessive or imbalanced aromatic amino acid fermentation may aggravate epithelial stress and disrupt mucosal homeostasis. Therefore, the value of low-protein diets lies in correcting excessive proteolytic fermentation, rather than completely suppressing microbial amino acid metabolism [39,84,85].

With adequate supplementation of limiting amino acids, such as lysine, methionine, threonine, tryptophan, valine, and isoleucine, moderate crude protein reduction can reduce hindgut protein fermentation while maintaining essential amino acid supply and growth performance. From the perspective of host–microbiota interactions, this strategy can optimize the intestinal substrate environment. It may shift microbial metabolism away from excessive proteolytic fermentation toward a more balanced fermentation profile, thereby reducing pro-inflammatory metabolic pressure and supporting epithelial barrier integrity and mucosal immune homeostasis. However, its limitations are clear. If amino acid balance, energy supply, fermentable carbohydrate availability, and ingredient digestibility are not properly controlled, excessive protein reduction may impair digestive enzyme activity, intestinal morphology, microbial fermentation capacity, and growth performance. Therefore, low-protein diets should be implemented as a precision feeding strategy. Their formulation should take into account standardized ileal digestible amino acid supply, the ratio of fermentable carbohydrates to protein, feed ingredient digestibility, and the physiological stage of piglets.

#### 7.5.2. Maternal Nutritional Programming: Early-Life Shaping of Microbiota and Intestinal Development

Maternal nutritional programming emphasizes regulation of the nutritional status of sows during gestation and lactation, thereby shifting the intervention window from the post-weaning period to prenatal and early postnatal stages and indirectly shaping the early microbiota and intestinal development of piglets. Sows can participate in shaping the early gut microbiota, barrier development, and mucosal immune maturation of piglets through vertical microbial transmission during parturition and through active components in milk, including microorganisms, immunoglobulins, milk-derived oligosaccharides, and metabolites [10,103]. Maternal diet, maternal microbiota, and milk composition can be regarded as upstream regulatory layers in the establishment of the piglet host–microbiota interaction network (Figure 3).

Experiments involving compound probiotic supplementation in sows have shown that maternal microecological intervention can reshape the colostrum metabolome, particularly pathways related to tryptophan metabolism and primary bile acid biosynthesis, while improving sow reproductive performance and offspring growth. These findings suggest that maternal interventions may establish transgenerational regulatory links among the maternal microbiota, mammary metabolism, and piglet intestinal development.

The concept of sensory-equivalent diets can be viewed as an extension of maternal nutritional programming during the weaning transition. Reduced feed intake after weaning is one of the important starting points that trigger host–microbiota interaction imbalance. Maintaining a degree of continuity in sensory characteristics, such as odor and taste, among the sow diet, creep feed, and weaning feed can reduce the adaptation barrier during the transition to solid feed and improve acceptance of weaning feed. This may help alleviate microbial disturbance, insufficient barrier repair, and growth restriction caused by low feed intake [10,59].

Further work is still needed to clarify the key regulatory windows and persistence of effects in maternal nutritional programming. It remains unclear whether different nutritional components have window-specific effects during gestation and lactation, whether the same additives produce differential effects under different basal dietary backgrounds, and how long their beneficial effects on piglet microbiota and intestinal health persist after weaning. Future studies should combine time-series sampling with sow-piglet paired analyses to clarify the windows of action, transmission pathways, and sustained effects of maternal interventions, thereby providing a more stable mechanistic basis for production application.

### 7.6. Integrated Evaluation of Network-Oriented Intervention Strategies

Overall, nutritional intervention strategies for weaned piglets should be evaluated within a hierarchical network-restoration framework rather than as isolated additive categories. Microbial community remodeling strategies, such as probiotics and FMT, mainly aim to restore ecological structure and colonization resistance. Metabolic microenvironment modulation strategies, including prebiotics, postbiotics, SCFAs, MCFAs, organic acids, and fermented feed, regulate substrate utilization, microbial fermentation, and metabolite availability. Host-directed strategies, including functional amino acids and phytogenic extracts, support epithelial barrier repair, mucus synthesis, antioxidant defense, and immune homeostasis. Systemic nutritional management strategies, such as low-protein diets and maternal nutritional programming, act on upstream dietary and developmental determinants of host–microbiota stability. Future studies should therefore move beyond evaluating growth performance and diarrhea incidence alone. They should integrate microbial composition, functional genes, metabolite profiles, epithelial barrier markers, mucosal immune indicators, and production outcomes to determine whether an intervention truly restores the disrupted host–microbiota network rather than temporarily improving a single phenotype.

Therefore, future studies should not use growth performance and diarrhea incidence as the only major endpoints. Instead, they should integrate multidimensional data, including microbial composition, functional genes, metabolic profiles, barrier-related markers, mucosal immune indicators, and production performance. This integrated evaluation would help determine whether a nutritional intervention truly promotes the restoration of host–microbiota interaction networks, rather than only improving a single short-term phenotype. A major bottleneck in the practical application of antibiotic-alternative strategies is their variable efficacy under production conditions. Probiotics, postbiotics, prebiotics, and functional nutrients often perform well in controlled trials. However, their effects may differ substantially among farms, herds, and production systems. This variation is not only related to product quality or additive type. It also reflects multiple confounding factors within the host–microbiota–environment network. First, the genetic background of piglets may influence the intestinal epithelial microenvironment, immune response thresholds, microbial colonization patterns, and responsiveness to nutritional or microbial interventions. Therefore, the same probiotic strain or postbiotic preparation may produce different outcomes in piglets of different breeds, genetic lines, or health status. Second, the baseline microbiota also affects intervention efficacy. In piglets with a stable and functionally redundant microbiota, exogenous strains may have difficulty colonizing or expressing their functions. In contrast, in piglets with severe dysbiosis, impaired barrier function, or high pathogen pressure, a single intervention is often insufficient to restore intestinal homeostasis. In such cases, dietary adjustment, environmental improvement, and management optimization may be required.

In addition, weaning age, mixing, transportation, temperature fluctuation, stocking density, hygiene conditions, pathogen exposure, and feed transition stress can all affect microbial succession, feed intake recovery, immune activation, and barrier repair. These production-related stressors may mask or even offset the biological effects of antibiotic alternatives. Therefore, future precision nutrition studies should move beyond the evaluation of average treatment effects. Greater attention should be given to the stratification of responders and non-responders. Intervention trials should also consider host genetic background, baseline microbiota structure, colonization resistance, diet composition, environmental stress load, and clinically relevant outcomes. This would help identify the specific conditions under which different antibiotic-alternative strategies are most likely to succeed.

## 8. Frontiers and Future Perspectives

Future research on piglet intestinal host–microbiota interactions should be organized according to a clear progression from mechanistic validation to precision intervention and finally to translational application. Although several promising directions have emerged, they do not have the same level of urgency or maturity. The most immediate priority is to move from correlation-based descriptions toward causal and standardized evaluation systems. On this basis, high-priority mechanistic and intervention studies should focus on precision microbiome regulation, gut–brain–microbiota communication, and microbiome-based mitigation of feed-derived stressors. Finally, industrial safety assessment and the translational value of pig models should be developed as application-oriented extensions of these mechanistic advances.

Although substantial progress has been made in describing microbial dysbiosis, barrier injury, inflammatory activation, and nutritional regulation in weaned piglets, several important knowledge gaps remain. First, the temporal and intestinal segment-specific dynamics of host–microbiota interactions are still incompletely understood. Most studies rely on fecal or single-time-point samples, which cannot fully reflect microbial and host responses in different intestinal segments during the rapid transition from suckling to weaning. Second, many reported associations among microbial taxa, metabolites, and host barrier or immune markers still lack causal validation. Third, the responses to probiotics, prebiotics, postbiotics, and functional nutrients are often context-dependent, but the host, microbial, dietary, and environmental factors that determine responder and non-responder phenotypes remain poorly defined. Future research should therefore prioritize longitudinal, multi-site, and mechanism-oriented studies that link microbial changes with functional metabolites; host signaling pathways; and clinically meaningful outcomes such as diarrhea incidence, feed intake recovery, and growth performance.

Future research should pay greater attention to the potential role of the gut microbiome in the detoxification and risk mitigation of feed contaminants. Feed-borne chemical contaminants, including mycotoxins, heavy metals, and other harmful substances, can impair intestinal barrier integrity, promote oxidative stress, and disturb gut microbial balance. Meanwhile, intestinal microorganisms may contribute to detoxification by reducing the bioavailability or toxicity of contaminants such as deoxynivalenol (DON) and aflatoxins through adsorption, biotransformation, or sequestration. Microecological detoxification strategies based on probiotics, postbiotics, and microbial enzyme preparations are expected to expand gut microbiome research from nutritional intervention and disease prevention to broader applications in feed safety, food safety, and sustainable animal production [49].

The role of the gut–brain axis in regulating animal welfare also deserves attention. Management factors such as the quality of human-pig interaction, weaning method, mixing stress, transport stress, temperature changes, and stocking density can jointly affect stress recovery, intestinal homeostasis, and production performance in piglets through the HPA axis, neuroendocrine signals, and gut microbial networks. This suggests that animal welfare management is not only a behavioral or feeding-management issue but should also be understood within the framework of host–microbiota interactions and microecological regulation. Future studies should therefore integrate behavioral indicators, stress hormones, microbial profiles, and intestinal barrier or immune parameters to clarify how welfare-related stressors reshape the gut–brain–microbiota axis in piglets [40].

Precision microbiome intervention is an important future direction for regulating intestinal health in piglets. Studies have found that current intervention experiments using probiotics, prebiotics, and plant bioactive compounds often show unstable effects, which are frequently related to differences in herd genetic background, initial microbial structure, weaning age, diet composition, and pathogen pressure. Chinese indigenous pig breeds and commercial pig breeds differ in barrier development, microbial composition, and metabolite profiles. Lactobacilli derived from local breeds such as Ningxiang pigs and Mashen pigs show certain host-adaptation advantages. These observations suggest that microecological intervention should shift from general additive use toward individualized regulation strategies based on host genetic background, microbial composition, and disease-status stratification [26,82].

Future studies should further clarify the optimal range and mechanism of crude protein reduction in weaned piglets. More attention should be paid to how different degrees of crude protein reduction, amino acid balance, protein source digestibility, fermentable carbohydrate supply, and weaning age jointly determine microbial composition, protein fermentation, nitrogen metabolism, barrier function, immune responses, and growth performance. Integrated metagenomics, metabolomics, nitrogen-flow analysis, and host transcriptomics may help identify the dietary conditions under which crude protein reduction shifts the microbiota away from harmful proteolytic fermentation without compromising amino acid supply or intestinal development. Such work will be important for developing low-protein, amino acid-balanced diets as precise host–microbiota regulatory tools rather than only environmental nitrogen-reduction strategies.

The translational value of pigs in human intestinal disease research warrants further exploration. Because pigs are more similar to humans than mice in intestinal structure, physiological function, mucosal immunity, and nutritional metabolism and because their gut microbiome shows high similarity to the human gut microbiome at the level of functional pathways, humanized microbiota pig models can be used as physiologically relevant large-animal platforms for studies of inflammatory bowel disease, metabolic syndrome, microbiome-based therapy, and related topics. The piglet weaning stress model also provides an important reference for analyzing relationships among early-life stress, microbial dysbiosis, barrier injury, and disease susceptibility.

From the methodological perspective, future research should move beyond correlation-based descriptions toward validation of causal mechanisms. Most current studies still rely mainly on parallel changes in microbial structure, metabolic profiles, and barrier indicators, and multi-omics analyses often remain at the level of association-network construction. Future work should establish mechanistic loops centered on “key strains–functional metabolites–host receptors/signaling pathways–barrier endpoints” and verify causal chains through germ-free or simplified microbiota models, targeted colonization, intestinal organoid co-culture, metabolite rescue, receptor blockade, and pathway knockdown [80,104]. In this context, organ-on-a-chip technology may provide a more controllable experimental system for mechanistic validation of host–microbiota interactions. By incorporating continuous perfusion, mechanical stimulation, epithelial barrier monitoring, and communication between multiple tissue modules, this technology can help overcome the limitations of conventional in vitro models in dynamic simulation. Multi-organ-on-a-chip systems are particularly useful for questions that cannot be fully addressed by isolated intestinal models, such as bile acid signaling between the gut and liver, inflammatory cascade responses between the gut and immune system, and neuroendocrine regulation along the gut–brain axis. Recent methodological advances also indicate that functional coupling between organ modules, sensor-based real-time monitoring, automated perfusion control, and standardized data collection are key directions for improving the physiological relevance and reproducibility of multi-organ-on-a-chip platforms [105]. These features are closely aligned with the future needs of piglet intestinal health research, in which the focus should gradually shift from single static endpoint measurements to dynamic and mechanism-oriented assessments of intestinal barrier function, microbial metabolic activity, and systemic host responses. However, organ-on-a-chip systems specifically designed for piglets are still at an early stage. Therefore, findings from human-derived or general biomedical chip models should be considered methodological references rather than direct in vivo evidence for weaned piglets.

Industrial application requires early incorporation of safety and standardization assessments. Before candidate strains are commercialized, whole-genome safety evaluation should be completed, with emphasis on screening for antimicrobial resistance genes, virulence genes, and mobile genetic elements. Their viability stability, colonization persistence, dose controllability, and population-level reproducibility should also be verified during pelleting, storage, transport, use in complex diets, and application in actual farm environments [13,23,89]. Safety evaluation should run through the entire process of strain screening, animal-model validation, and mechanistic research, rather than serving only as a final review before product launch.

In addition, endpoints for intestinal barrier evaluation require further standardization. Although current studies widely use indicators such as villus morphology, tight-junction proteins, permeability markers, the mucus layer, sIgA, and inflammatory factors, inconsistencies in sampled intestinal segments, time points, detection methods, and statistical criteria limit comparability among studies [103]. Future work should build a stratified evaluation framework covering structural barriers, functional barriers, immune barriers, microbial barriers, and production outcomes and should set core endpoints in multi-omics studies to form a more stable and comparable chain of mechanistic evidence.

Overall, future research should follow a staged framework. The first stage should establish causal mechanisms and standardized evaluation systems. The second stage should use this foundation to develop precision microbiome interventions, clarify gut–brain–microbiota regulation, and expand microbiome research to feed-contaminant mitigation. The third stage should focus on industrial safety, farm-scale validation, and translational model development. This logical progression may help transform piglet host–microbiota research from descriptive characterization into mechanism-guided, production-relevant, and translationally valuable strategies for improving intestinal health.

## 9. Conclusions

Piglet intestinal health is not determined by a single pathogen or a single barrier indicator but reflects the coordinated status of the gut microbiota, epithelial barrier, mucosal immunity, microbial metabolites, and stress-related regulation. During early life, the piglet gut microbiota is gradually established under the influence of maternal microbial transmission, milk-derived nutrients, environmental exposure, dietary transition, and host selection. Stable early colonization supports barrier development, immune maturation, and metabolic adaptation, whereas weaning-associated dietary, environmental, and microbial stressors can disrupt this balance and increase the risk of diarrhea, inflammation, and impaired growth.

Gut microorganisms participate in regulating host barrier repair, immune tolerance, inflammatory thresholds, and metabolic homeostasis through signaling molecules such as SCFAs, tryptophan metabolites, bile acid derivatives, and amino acid-derived metabolites. The host reciprocally shapes microbial composition, spatial distribution, and functional status through factors including mucins, antimicrobial peptides, sIgA, intestinal alkaline phosphatase, and genetic background. Therefore, future research on piglet intestinal health should move beyond describing microbial composition alone and toward mechanistic chain analysis of “microbiota–metabolites–host pathways–barrier phenotypes.” Integrated multi-omics, culturomics, intestinal organoid models, simplified microbiota colonization, and targeted intervention experiments will become important technical routes for clarifying causal relationships and key regulatory nodes.

At the application level, probiotics, prebiotics, postbiotics, fecal microbiota transplantation, sow nutritional regulation, and functional nutrients provide important approaches for improving intestinal health in weaned piglets. However, these strategies should not be viewed as unrelated alternatives. They should be evaluated according to their specific roles in microbial community remodeling, metabolite production, barrier repair, immune regulation, and production performance. It should also be noted that their efficacy may vary under practical conditions. This variation is influenced by multiple factors, including host genetic background, baseline microbiota status, colonization resistance, diet composition, and environmental stress. Future studies should further integrate multi-omics analyses, culturomics, intestinal organoid or organ-on-a-chip models, targeted intervention trials, and responder/non-responder stratification. Such integrated approaches will help clarify key causal mechanisms and support the development of safer, more stable, and more precise antibiotic-alternative strategies for pig production.

## Figures and Tables

**Table 1:** Search strategy and number of records retrieved from each database.

Search Topic	Search Terms	Records Retained After Screening
Piglet gut microbiota and weaning	“weaned piglets” or “weaning piglets” and “gut microbiota” or “intestinal microbiota” or “microbiome”	20
Host–microbiota interaction	“piglets” and “host-microbiota interaction” or “host-microbe interaction”	18
Microbial succession and colonization	“piglets” and “microbial colonization” or “microbiota succession” or “early-life microbiota”	13
Intestinal barrier and mucosal immunity	“weaned piglets” and “intestinal barrier” or “tight junction” or “mucosal immunity” or “sIgA”	29
Microbial metabolites	“piglets” and “SCFAs” or “tryptophan metabolites” or “bile acids” or “amino acid metabolism”	24
Gut–brain axis	“piglets” and “gut-brain axis” or “neuroendocrine” or “HPA axis” or “cortisol”	6
Omics technologies	“weaned piglets” and “metagenomics” or “metabolomics” or “single-cell transcriptomics” or “multi-omics”	14
Nutritional interventions	“weaned piglets” and “crude protein reduction” or “low-protein diet” or “reduced-protein diet” and “probiotics” or “prebiotics” or “postbiotics” or “synbiotics” or “fecal microbiota transplantation” or “functional amino acids”	31

Note: The number of records retrieved refers to the results obtained from each database using the indicated search terms on 31 May 2026. Records retained after screening refer to studies finally included in the narrative synthesis for each thematic category. Because some studies addressed more than one topic, the same article could be assigned to more than one category.

## Data Availability

No new data were created or analyzed in this study. Data sharing is not applicable to this article.

## Abbreviations

The following abbreviations are used in this manuscript:

mGWAS	Microbiome-wide genome association studies
SCFAs	Short-chain fatty acids
AHR	Aryl hydrocarbon receptor
HIF-1α	Hypoxia-inducible factor 1-alpha
GABA	Gamma-aminobutyric acid
DON	Deoxynivalenol
ZEN	Zearalenone
IAP	Intestinal alkaline phosphatase
MAMPs	microbe-associated molecular patterns
ETEC	Enterotoxigenic *Escherichia coli*
FMT	Fecal microbiota transplantation
LPS	Lipopolysaccharide
MCFAs	Medium-chain fatty acids
HPA	hypothalamic–pituitary–adrenal
5-HT	5-hydroxytryptamine
cAMP	Cyclic adenosine monophosphate
STb	heat-stable enterotoxin b
Th17	T-helper cell 17
sIgA	Secretory immunoglobulin A
FOS	Fructo-oligosaccharides
GOS	Galacto-oligosaccharides
XOS	Xylo-oligosaccharides
MOS	Mannan-oligosaccharides
DAO	Diamine oxidase
